# Single-cell RNA-seq with spike-in cells enables accurate quantification of cell-specific drug effects in pancreatic islets

**DOI:** 10.1186/s13059-020-02006-2

**Published:** 2020-05-06

**Authors:** Brenda Marquina-Sanchez, Nikolaus Fortelny, Matthias Farlik, Andhira Vieira, Patrick Collombat, Christoph Bock, Stefan Kubicek

**Affiliations:** 1 CeMM Research Center for Molecular Medicine of the Austrian Academy of Sciences, Lazarettgasse 14, AKH BT 25.3, 1090 Vienna, Austria; 2 Department of Dermatology, Medical University of Vienna, 1090 Vienna, Austria; 3 Université Côte d’Azur, CNRS, Inserm, iBV, Nice, France; 4 Department of Laboratory Medicine, Medical University of Vienna, 1090 Vienna, Austria

## Abstract

**Background:**

Single-cell RNA-seq (scRNA-seq) is emerging as a powerful tool to dissect cell-specific effects of drug treatment in complex tissues. This application requires high levels of precision, robustness, and quantitative accuracy—beyond those achievable with existing methods for mainly qualitative single-cell analysis. Here, we establish the use of standardized reference cells as spike-in controls for accurate and robust dissection of single-cell drug responses.

**Results:**

We find that contamination by cell-free RNA can constitute up to 20% of reads in human primary tissue samples, and we show that the ensuing biases can be removed effectively using a novel bioinformatics algorithm. Applying our method to both human and mouse pancreatic islets treated ex vivo, we obtain an accurate and quantitative assessment of cell-specific drug effects on the transcriptome. We observe that FOXO inhibition induces dedifferentiation of both alpha and beta cells, while artemether treatment upregulates insulin and other beta cell marker genes in a subset of alpha cells. In beta cells, dedifferentiation and insulin repression upon artemether treatment occurs predominantly in mouse but not in human samples.

**Conclusions:**

This new method for quantitative, error-correcting, scRNA-seq data normalization using spike-in reference cells helps clarify complex cell-specific effects of pharmacological perturbations with single-cell resolution and high quantitative accuracy.

## Introduction

Recent advances in single-cell transcriptome profiling have enabled the comprehensive characterization of cell populations in multiple tissues, resulting in initial drafts of mouse and human cell atlases [1–4]. To date, these atlases focus primarily on the static cell composition in tissues, while there is as yet little information on the dynamic responses of individual cells to stimuli in a whole-tissue setting. Such response dynamics are of particular interest in pancreatic islets of Langerhans, a tissue composed of multiple endocrine cell types defined by their marker hormones glucagon (alpha cells), insulin (beta cells), pancreatic polypeptide (PP cells), somatostatin (delta cells), and ghrelin (epsilon cells). Cell-type-specific transcriptomes are established during development, yet even fully mature islet cells retain the ability to alter their cellular identity by dedifferentation and transdifferentiation. Furthermore, islet cells respond transcriptionally to the blood glucose levels they control through their secreted hormones and to drugs that target the glucose sensing and hormone secretion pathways. Importantly, all of these processes are dependent on an intricate paracrine and endocrine crosstalk between the different cell subtypes, requiring their study at the whole-tissue level.

In adult islets, most cells express a single hormone at the protein level, and only approximately 1% of cells are being described as polyhormonal [5–8]. Whether this is also true on the transcriptome level is currently unclear. Different single-cell transcription studies [9–23] by RNA-seq, RNA-PCR, and RNA-FISH reached different conclusions regarding the levels of polyhormonality. While some studies conclude that the majority of endocrine cells express more than one hormone [10, 24, 25], others find that islet cells predominantly are monohormonal also on the transcript level [26]. These discrepancies may reflect different sensitivities and detection limits as well as technical limitations such as RNA cross-contamination or the inadvertent analysis of cell doublets instead of single cells.

The question of polyhormonality is of particular importance during dedifferentiation and transdifferentiation processes that are thought to often proceed through a stage where cells coexpress multiple hormones and progenitor markers [7, 27–33]. These processes can be induced genetically through the aberrant expression or the ablation of cell-type-specific master regulatory transcription factors in animal models. Most notably, alpha cells have been shown to undergo transdifferentiation to beta cells following overexpression of PAX4 [30] and ablation of ARX either alone or in combination with DNMT1 [27, 28]. Moreover, beta cell-specific knockout of FoxO1 is a well-established dedifferentiation model, resulting in reduced beta cell mass and adoption of alpha cell fate by a subset of beta cells [33]. Direct translation of these genetic animal models to primary human islets is challenging due to inefficient genome editing and limited functional timespan of intact islets in vitro. Only recently, it was shown that human alpha and PPY cells can be converted to insulin-producing cells by overexpression of both PDX1 and MAFA [34].

Alterations of pancreatic endocrine cell identity have also been achieved with pharmacologic agents, which, compared to genetic methods, promise faster kinetics, dose-responsiveness, species-independence, and potential clinical utility. For example, a small-molecule FOXO inhibitor [35] has been shown to convert delta cells to insulin-producing cells following near-complete beta cell ablation [36]. Moreover, we previously reported that the small-molecule artemether impairs alpha cell identity and induces insulin expression, resulting in improved glucose tolerance in diabetes models [37]. Recent studies have demonstrated that artemether indeed improves glucose tolerance in multiple in vivo models [37–42]. Similarly, GABA has been reported to improve glucose homeostasis by alpha cell transdifferentiation or beta cell proliferation [43–52]. However, the mechanisms of artemether and GABA have been controversial, as two recent studies have not detected an increase in alpha cell-derived beta cells using lineage tracing models [8, 41].

To clarify cell-type-specific drug effects in primary islets requires to quantify low-level induction of cell type foreign hormones with high accuracy, robustness, and sample-to-sample comparability. In particular, any sample carryover or cross-contamination that occurs in droplet-based single-cell RNA-seq needs to be effectively corrected for. To that end, we develop a method that combines standardized reference cells as spike-in controls with a computational decontamination algorithm. Applying our method to ex vivo pancreatic islet samples, we obtain 24,729 mouse and 82,463 human single-cell islet transcriptomes in response to drug treatment of undissociated tissue samples. Using our method, we observe dramatically lower numbers of polyhormonal cells than based on the uncorrected analysis, in line with previous observations on the protein level. We further show that a small-molecule FOXO inhibitor can induce beta cell dedifferentiation ex vivo, and we observe that artemether consistently increases the expression of insulin and other beta cell genes in a subset of alpha cells.

## Results

### Reference cells as spike-in controls identify contamination in single-cell RNA-seq

**Fig. 1:**
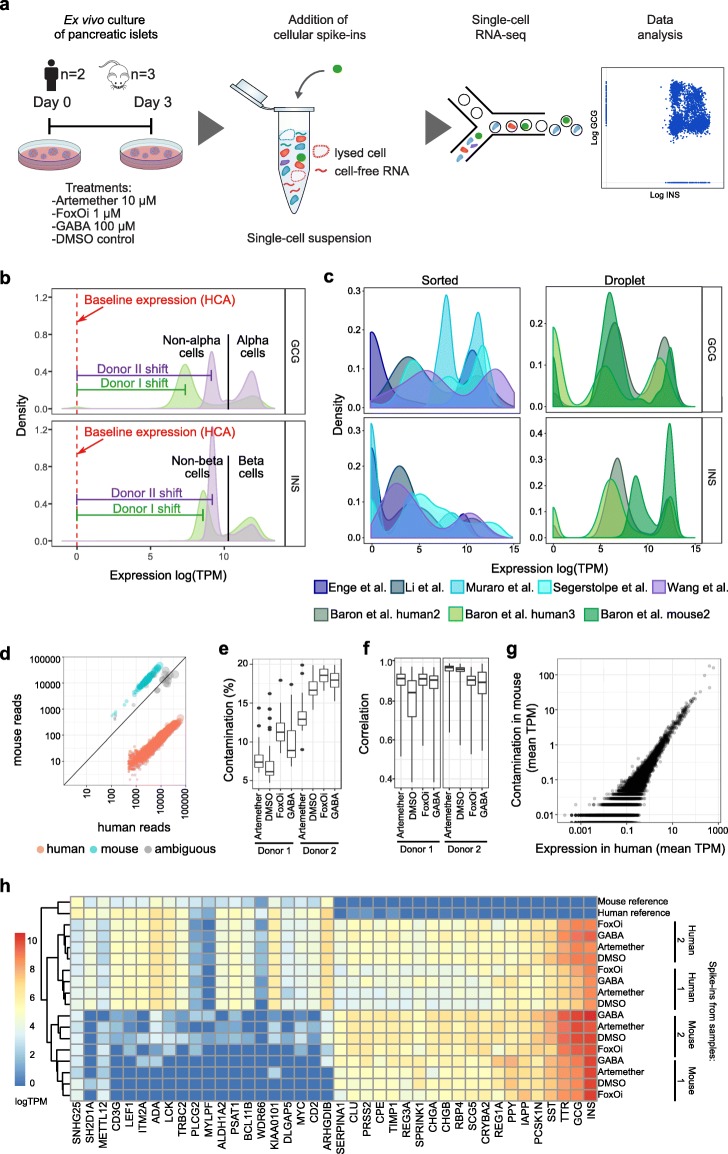
Spike-ins enable assessment of contamination in scRNA-seq experiments. **a** Islet treatment and sample preparation scheme. **b** Expression (log TPM) of insulin and glucagon in all cells of DMSO-treated islets from human donors I and II. Horizontal lines indicate the shifts of the non-alpha and non-beta cells from non-expressing levels. Red dotted line: baseline expression for non-islet cells as reported in the Human Cell Atlas (HCA). **c** Expression of insulin and glucagon in external datasets. TPM and RPKM values reported in each dataset were log transformed. **d** Alignment of reads to both the human and mouse genomes to identify spike-in reference cells, here mouse spike-ins in a human sample (DMSO, donor II). **e** Contamination of mouse spike-ins in human samples. Contamination is quantified as the percentage of reads aligned to the human genome in mouse spike-ins. **f** Correlation of the contamination profile within the mouse spike-in cells in human samples. **g** Average contamination in mouse spike-ins (*y*-axis) versus average expression in the sample (human pancreatic cells, *x*-axis) shown for sample DMSO, human donor II. Spearman correlation *R* = 0.888. **h** Average expression (log TPM) of genes with the largest difference in mouse and human spike-ins, and external references

To systematically assess the effect of drug treatments on primary pancreatic islets with quantitative cell-type-specific resolution, we exposed intact human and mouse islets to 10 μM artemether, 100 μM GABA, 1 μM FoxO inhibitor AS1842856 (FoxOi), and control DMSO for 72 h ex vivo (Fig. 1a). After that period, we dissociated islets, filtered for single cells, and performed single-cell transcriptome analysis on the 10X Chromium platform. Following alignment and initial quality control, we obtained a total of 142,165 transcriptome profiles.

Analyzing the resulting human transcriptomes, we observed striking sample-specific differences in glucagon (GCG) and insulin (INS) expression across all cell types between replicates (Fig. 1b). In addition, we observed surprisingly high levels of expression for both GCG (3.0% and 6.1% of all reads in replicates I and II, respectively) and INS (6.5% and 4.5%) in all cells, and no INS-negative cells were observed (Fig. 1b). Similar patterns are present in most published droplet-based scRNA-seq datasets of pancreatic islets (Fig. 1c). In contrast, hormone-negative cell populations were detected in some scRNA-seq studies based on sorting cells to individual wells, although also there the majority of islet cells have detectable levels of both INS and GCG. The high study dependence and low fraction of monohormonal cells indicates potential technical biases in the scRNA-seq results.

Inclusion of internal standards is widely appreciated for quality assessment, normalization, calibration, and quantitation of analytical data [53]. Such internal standards should be spiked in early during the sample processing workflow and have highly similar, yet clearly discriminable properties to the analytical samples. For single-cell transcriptomics, ideal internal standards are therefore well-characterized homogenous cell populations. We chose to use two cell lines, mouse 32D and human Jurkat cells, as internal standards, both of which we characterized by RNA-seq. Methanol-fixed cells (~ 5% of all cells) were spiked into all samples shortly before droplet formation, and thus prior to processing, sequencing, and bioinformatics analyses as part of the entire dataset. We found that cross-species spike-ins (here mouse 32D cells) provide particularly clean reference points when aligned to a combined human/mouse reference genome, for two reasons. First, they were easily separable from other cells (here: human islet and spike-in) by the ratio of human/mouse reads per cell (Fig. 1d and Additional file 1: Fig. S1). Second, counting the number of cross-aligned reads (here: human reads found in mouse cells) provided a straightforward way of evaluating sample-specific biases.

Measuring contamination as the percentage of reads that aligned to the human reference in mouse spike-in cells (Fig. 1e), we observed a high, sample-specific, level of contamination in both samples (medians 8.1% and 17.4% in samples from human donors I and II, respectively), with a maximum of 19.9% in sample I. However, the contamination profile was highly similar within the cells of each sample (Fig. 1f). This human contamination in mouse spike-ins was highly correlated with the average expression in human cells (Fig. 1g), suggesting that the contamination was indeed derived from islet RNA. To identify affected genes, we next compared mouse and human spike-ins in human islet samples to mouse and human spike-ins sequenced in isolation as external reference, all aligned to the human genome (Fig. 1h). Both human and mouse spike-ins from islet samples showed strong expression of major islet marker genes such as INS, GCG, TTR, SST, PPY, IAPP, and REG1A. Given that expression of these genes was virtually absent from reference cells, it can be attributed to contamination.

Likely the contamination was found in the medium or buffer the cells are resuspended in, in the form of cell-free RNA originating from dying cells that were enclosed in droplets during processing. Possibly cell lysis may occur during the single-cell transcriptomics workflow during incubation of the cell suspension with the master mix providing reagents for the reverse transcription step prior to droplet generation. Importantly, both empirical and theoretical considerations excluded the alternative hypothesis that contamination occurs between samples during sequencing through index switching [54]. Empirically, we observed low overlap in barcodes between experiments run on the same lane (Additional file 1: Fig. S2). Theoretically, all contaminated cells (barcodes) in a given sample would require a corresponding cell (barcode) in another sample that was processed on the same lane and contains all contaminating genes. This is highly unlikely given that only a small fraction of all barcodes is labeled as cells.

### Spike-in reference cells enable accurate computational correction of cell signatures

We model contamination as a transcriptional signature that is added to the transcriptional signature of each cell. While the signature of contamination is highly similar across cells within each sample, the extent to which this profile is added is specific to each cell (Fig. 1e). Decontamination thus requires the estimation of two variables. First, the extent of total contamination of each cell, quantified as the fraction of contaminating reads (*f*_c_). Second, the extent to which each gene contributes to the contamination in each sample, i.e., the transcriptional signature of contamination (*S*_c_). Estimation of these factors is difficult in classical single-cell RNA-seq experiments where only the final, contaminated profile of each cell is observed. However, both variables can be estimated from spike-in cells, where contaminated expression profiles can be compared to clean reference profiles, which were obtained by sequencing spike-ins separately (in the absence of potentially contaminating cells). Of particular advantage are cross-species spike-ins, where cross-species aligned reads enable straightforward quantification of contamination signature and fraction in those spike-ins (Fig. 1g). For example, reads aligned to the human genome in a mouse cell are likely to arise from contamination.

**Fig. 2:**
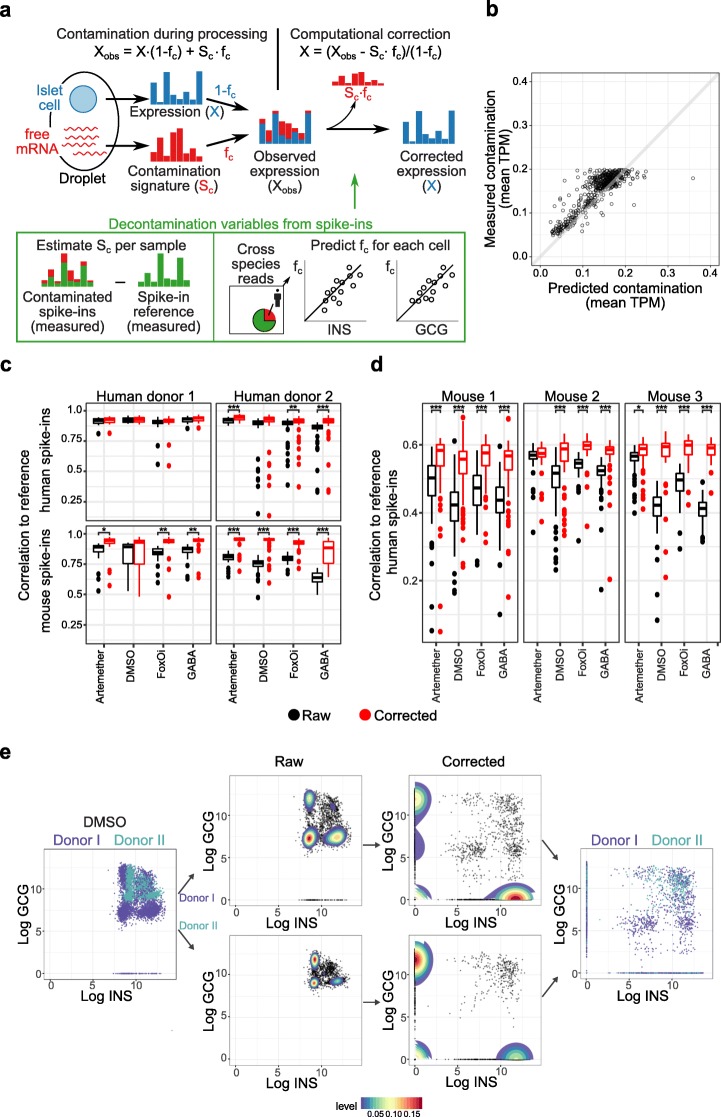
Accurate computational correction of cellular signatures based on spike-in cells. **a** Mouse spike-ins are used to estimate the fraction of contamination *f*_c_ and the signature of contamination *S*_c_. **b** Predicted versus measured *f*_c_. **c**, **d** Pearson correlation of raw (black) and corrected (red) values of the spike-ins in the human and mouse samples to the external reference spike-in transcriptome. **e** Flow diagram of data correction of DMSO samples from donors I and II. Left to right: 1—Scatter plot of raw INS and GCG from human donors I and II. 2 and 3—Density plots of donors separately of raw and corrected data. 4—Scatter plot of corrected INS and GCG from human donors I and II. ***p < 0.0001, **p < 0.001, *p < 0.005

Based on the above rationale, we devised a computational decontamination procedure based on spike-in cells to correct expression data of all cells (Fig. 2a). First, we estimated the *S*_c_ in each sample by comparing contaminated mouse spike-ins to clean references. Next, we predicted the fraction of contamination for all cells. To do so, we relied on mouse spike-ins, where *f*_c_ was calculated as the fraction of cross-species aligned reads. Based on these ground truth *f*_c_ values in mouse spike-ins, we fitted linear models that learn to predict *f*_c_ based on the expression of the most contaminating genes. Next, these models were applied to predict *f*_c_ in all human cells. Finally, *S*_c_ scaled by *f*_c_ was subtracted from the expression profile of all cells.

To evaluate our correction procedure, we first assessed the accuracy of our f_c_ predictions using 3-fold cross validation within mouse spike-ins. This assessment showed high prediction performance (Fig. 2b, *R* = 0.72). Next, we correlated the corrected values of mouse and human spike-ins to clean external references. In all cases, corrected values correlated more strongly with external references than did the raw data. This evaluation was done in cross-validated mouse as well as human data unseen by the predictor (Fig. 2c). Similar high performance was observed when decontaminating mouse cells based on human spike-ins (Fig. 2d).

After decontamination, hormone reads were only present in endocrine cells and not in all cell populations (Additional file 1: Fig. S3a). Also, our decontamination method showed better performance when compared to other correction methods [55] (Additional file 1: Fig. S3b, Additional file 2: Table S1).

Altogether, these analyses demonstrate that cellular spike-in controls not only are powerful tools to detect sample contamination with cell-free RNA, but also enable highly accurate correction of contaminated data (Fig. 2e). Importantly, spike-ins require marginal sequencing resources as they only constitute a small fraction of all cells analyzed.

### Machine learning enables marker-free assignment of cell types in pancreatic islets

**Fig. 3:**
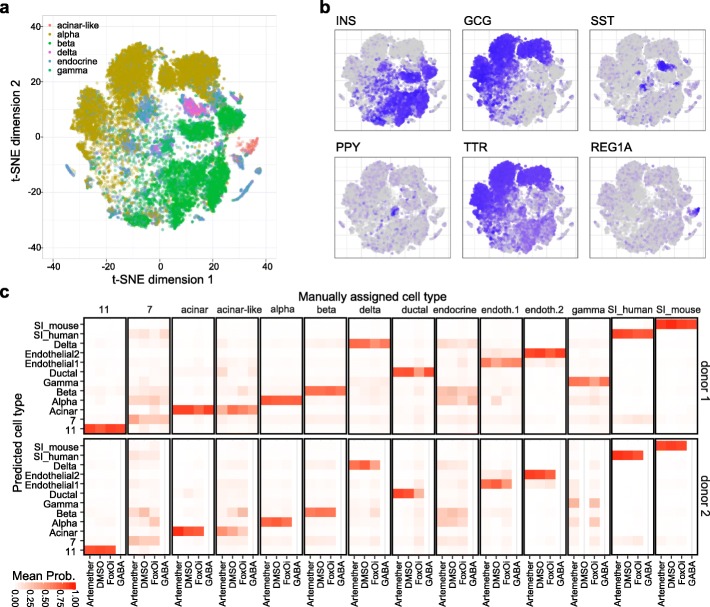
Cell type assignment. **a** t-SNE plot of endocrine human cells. **b** Expression of marker genes on t-SNE plot. **c** Cell type probability predicted for each cell not used in training in human samples. Cell type reassignment based on predictions was only done for those cells not previously assignable, here labeled “endocrine”

Based on our contamination-corrected expression data, we next sought to identify cell types to assess cell-type-specific effects of drug treatment. To identify islet cell subpopulations, we initially performed principal component analysis followed by t-distributed stochastic neighborhood embedding (t-SNE) on corrected gene expression data (Additional file 1: Fig. S4). We identified clusters of non-endocrine acinar, ductal, endothelial, and immune cells. In addition, we identified same-species spike-ins by their correlation to reference transcriptomes. Endocrine cell types were not well separated in this initial analysis. We therefore repeated clustering and t-SNE analysis separately for endocrine cells (Fig. 3a) and observed improved resolution but still no clear separation (Fig. 3b).

We then employed a machine learning-based approach, which was trained on unambiguously assignable cells, to predict cell type of difficult to assign cells. First, we assigned clear representatives of alpha, beta, gamma, and delta cells solely based on high expression of insulin, glucagon, somatostatin, or pancreatic polypeptide. In addition, a cluster expressing high levels of REG1A was assigned as “acinar like” in human samples (Fig. 3b). We next trained a classifier to predict cell type from the transcriptome based on these clear representatives of each cell type, which were first subsampled to reduce class imbalance. Importantly, cell type defining hormones were removed from the transcriptomes. The trained classifier was then applied to all cells not used in training, demonstrating the high prediction performance (Fig. 3c and Additional file 1: Fig. S5a). Computationally predicted class was then used to assign cell types to only those cells that were not assignable based on marker genes (labeled “endocrine” in Fig. 3c and Additional file 1: Fig. S5a). Since we are interested in studying cell type identities, a heterodoublet between two different cell types could have an impact on our analysis. Given that there was no clear cutoff for doublets removal based on the classical cutoffs of nGene or nUMI, we performed a cell-type-specific filtering based on cell type predictions and nGenes (Additional file 1: Fig. S6 and Additional file 3: Table S2).

Following assignment of single cells to cell types, we first analyzed the ratios of different cell types compared to the total population (Additional file 1: Fig. S5b-c). Overall, we observed strong donor-to-donor differences, which dominated over the effects of drug treatment with FoxOi, artemether, and GABA. To evaluate cell-type-specific drug effects, we calculated relative cell-type-specific gene expression changes by comparing compound treatment to donor-matched DMSO controls (Additional file 4: Table S3).

### Pharmacological inhibition of FoxO induces islet cell dedifferentiation in vitro

**Fig. 4:**
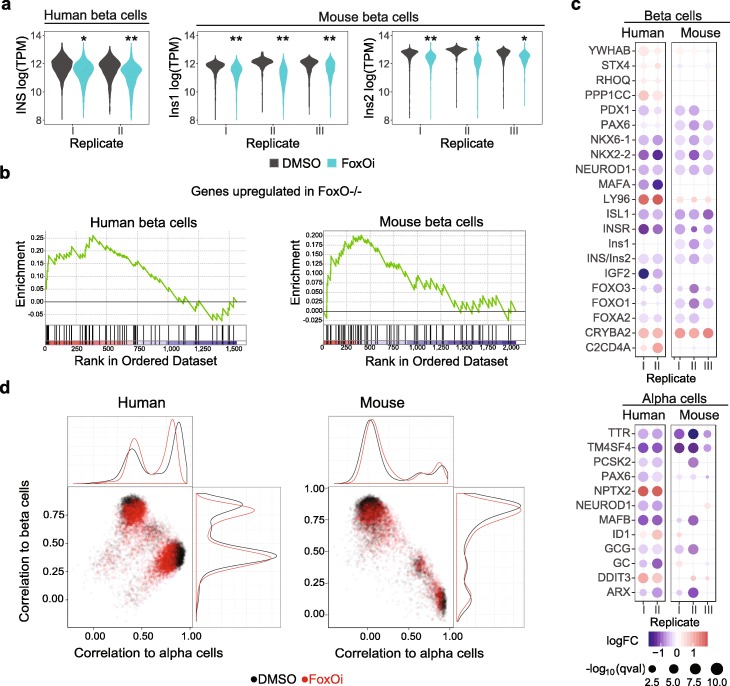
FoxOi induces dedifferentiation in alpha and beta cells of human and mouse islets. **a** Insulin expression in beta cells from human (INS) and mouse (Ins1 and Ins2) islets treated with 1 μM FoxOi for 72 h (*p < 10^−10^, **p < 10^−45^). **b** GSEA with the set of upregulated genes in triple Foxo knockout mice (FoxO1^−/−^, FoxO3a^−/−^, and FoxO4^−/−^) [57] in beta cells from human and mouse islets treated with 1 μM FoxOi for 72 h. **c** Gene expression changes in alpha (top) and beta (bottom) cells from human and mouse islets treated with 1 μM FoxOi for 72 h compared to DMSO treatment. **d** Correlation of alpha and beta cell gene expression from human and mouse islets treated with 1 μM FoxOi for 72 h to an alpha or beta cell gene signature set

We first analyzed the effects of the FoxOi on islet cells, to evaluate whether the compound can model beta cell dedifferentiation in vitro. In line with genetic models of FoxO loss [33, 56, 57], the FoxOi caused a reduction of insulin expression in mouse beta cells that we also observed in human beta cells (Fig. 4a, Additional file 5: Table S4).

To analyze whether transcriptome-wide changes reflected a true dedifferentiation event, we used gene set enrichment analysis (GSEA) to compare expression changes in beta cells treated with FoxOi to known beta cell dedifferentiation signatures [56]. Genes with increased expression in a triple FoxO KO mouse model were upregulated upon FoxOi treatment in mouse and human islets (Fig. 4b). Important beta cell transcription factors such as NEUROD1, ISL1, NKX6-1, NKX2-2, FOXA2, MAFA, PDX1, and FOXO1 were downregulated (Fig. 4c). Consistent with the genetic models of FOXO loss, we identified LY96 and immature beta cell markers CRYBA2 and C2CD4A to be upregulated in beta cells after FoxOi treatment. GSEA revealed that the main pathways associated with the downregulated genes in FoxOi-treated beta cells were “regulation of gene expression in beta cells,” “pancreatic secretion,” and “type II diabetes” (Additional file 6: Table S5).

Interestingly, we also observed a loss of alpha cell identity in response to FoxOi treatment. Glucagon, as well as the master transcription factor ARX, and other alpha cell-specific genes such as GC, NEUROD1, TTR, PAX6, PCSK2, MAFB, and TM4SF4 were downregulated (Fig. 4c).

In order to validate the loss of alpha cell and beta cell identity in an unbiased approach, we correlated gene expression signatures of FoxOi-treated cells to gene sets comprised of non-hormone alpha and beta cell markers (Fig. 4d). Thus, we obtained a value of correlation to an alpha or beta cell signature gene set for each single cell. In both mouse and human islets, FoxOi induced a loss of both alpha and beta cell identity and a shift in both cell populations to less committed gene expression signatures.

### A subset of alpha cells in islets treated with artemether upregulate insulin and beta cell markers

**Fig. 5:**
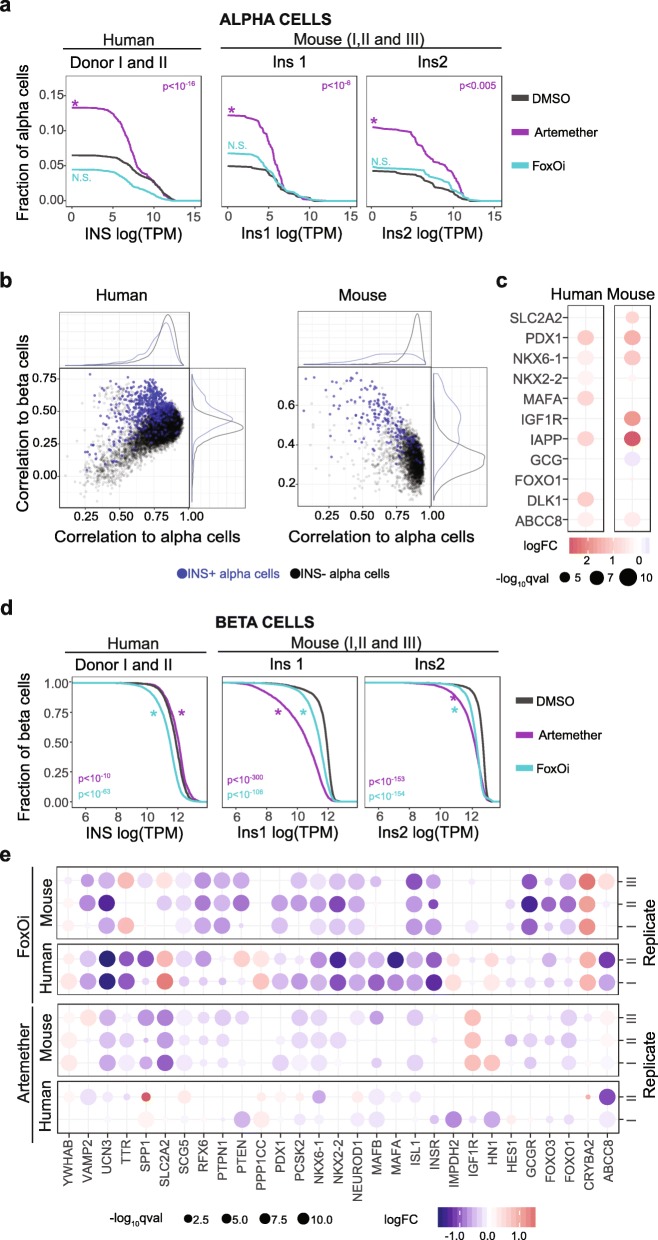
Artemether upregulates insulin in a subset of mouse and human alpha cells while effects in beta cells are species dependent. **a** Inverse cumulative distribution of insulin expression in assigned alpha cells from human (INS) and mouse (Ins1 and Ins2) islets treated with 1 μM FoxOi or 10 μM artemether or DMSO for 72 h. Plotted is the fraction of alpha cells that express insulin to a higher level as indicated on the *x*-axis. **b** Correlation of gene expression signatures in alpha cells without (Ins^−^) and with (Ins^+^) detectable insulin expression alpha or beta cell gene signature sets. **c** Gene expression changes of Ins^+^ alpha cells relative to Ins^−^ alpha cells. **d** Inverse cumulative distribution of insulin expression in beta cells from human (INS) and mouse (Ins1 and Ins2) islets treated with 1 μM FoxOi or 10 μM artemether or DMSO for 72 h. **e** Gene expression changes in human and mouse beta cells treated with 10 μM artemether or 1 μM FoxOi compared to DMSO

We next analyzed the effects of artemether on alpha cells based on the scRNA-seq data. In DMSO-treated controls, 1–6% of mouse and human alpha cells express detectable levels of insulin (4–6% Ins1, 0.7–3.3% Ins2, 1.3–4.3% INS). Following 10 μM artemether treatment for 72 h, we observed a consistent increase in the population of insulin-expressing alpha cells by approximately 3-fold in human islets and 2-fold in mouse islets (Fig. 5a, Additional file 1: Fig. S7a and Additional file 7: Table S6). This effect was also observed in a third human human donor treated with artemether for 72 h, but not for 36 h (Additional file 1: Fig. S8a). This increase was consistently detected in cells predicted to be alpha cells with > 50% probability for both human and mouse samples, ensuring that these cells should be considered as alpha cells rather than any other endocrine cell type (Additional file 1: Fig.S7b-c). This effect was observed independently of the insulin/glucagon ratio in islets from the different donors (Additional file 1: Fig. S7d). Importantly, treatment with FoxOi failed to increase the fraction of alpha cells that express insulin, suggesting an artemether-specific effect not linked to general dedifferentiation (Fig. 5a and Additional file 1: Fig. S7A).

We further characterized insulin-positive alpha cells by comparing their transcriptomes to all other alpha cells (Additional file 8: Table S7). In order to assess whether the increase in insulin expression was a reflection of loss of alpha cell identity and a possible induction of beta cell identity, we tested the correlation to gene signatures specific to alpha cells or beta cells, excluding cell type defining hormones (as above). Alpha cells that expressed insulin lost alpha cell identity and gained relevant aspects of beta cell identity compared to insulin-negative alpha cells (Fig. 5b). In human, the beta cell-specific genes IAPP, DLK1, ABCC8, PDX1, MAFA, NKX6-1, and NKX2-2 were increased and the alpha cell-specific genes GCG, ARX, and TTR were decreased, in line with a more general loss of alpha cell identity (Fig. 5c). The main upregulated pathways were “insulin secretion” and “insulin signaling.” In mouse, the beta cell-specific genes Ins1, Igf1r, Pdx1, Nkx6-1, Nkx2-2, Iapp, Foxo1, Abcc8, and Slc2a2 were all upregulated, corresponding to an increase in “regulation of gene expression in beta cells” and “beta cell development”-specific gene sets (Additional file 9: Table S8) [58, 59].

We also observed this induction of insulin/glucagon double positive cells likely arising from alpha cells in artemether-treated islets from a fourth donor with Drop-seq as an alternative technology to capture single-cell RNA expression (Additional file 1: Fig. S9) [60].

### The effect of artemether on beta cells in pancreatic islets is species-specific

We next analyzed the effects of artemether on beta cells. In mouse beta cells, artemether caused a strong decrease of insulin expression, in line with an earlier report that suggested the drug induces beta cell dedifferentiation [8] (Fig. 5d).

To confirm that these changes reflect a true dedifferentiation event, we compared the gene expression signatures of artemether-treated beta cells to the known transcriptomes of dedifferentiated beta cells using GSEA (Additional file 1: Fig. S10a). In mouse beta cells, artemether-induced gene expression changes correlated with those induced by FoxOi (Additional file 1: Fig. S10b, *R* = 0.518). With both compounds, we observed downregulation of genes in the insulin secretion, glucagon signaling, and FoxO signaling pathways such as Ucn3, Nkx6-1 Pcsk2, and FoxO1 (Fig. 5e).

In contrast to our observations for the mouse samples, beta cells isolated from human islets treated with artemether showed no reduction, and indeed a small increase, in insulin expression compared to DMSO-treated controls (Fig. 5d). Insulin expression was also not decreased in the third human donor at 36 nor 72 h (Additional file 1: Fig. S8b). This species specificity was in contrast to the effect of FoxOi, which caused insulin downregulation both in mouse and in human beta cells. In line with this difference between artemether and FoxOi treatment, the overall correlation of gene expression changes in human beta cells was found to be weaker between the two compounds (Additional file 1: Fig. S10b, *R* = 0.263). While FoxOi downregulated key beta cell genes including NKX2-2, PDX1, FOXA2, MAFA, and INSR, expression of these genes was mostly unaltered in artemether-treated human beta cells (Fig. 5e).

Finally, we compared drug effects across species by matching orthologous genes between mouse and human beta cells. We observed that the effects of the FoxOi were weakly correlated between species (Additional file 1: Fig. S10c, *R* = 0.296) whereas no correlation was observed for artemether effects in mouse and human beta cells (*R* = 0.129). While artemether downregulated key beta cell genes INS1/2,SLC2A2, ISL1, GCGR, UCN3, and SCG5 in mouse beta cells, the expression of these genes remained unchanged in human beta cells. In addition, many genes changed discordantly, for example artemether downregulated SPP1 in mouse beta cells, whereas it upregulated its expression in human beta cells, and vice versa for IGF1R. These data indicate that FoxOi effects are conserved in mouse and human beta cells, whereas artemether causes more species-dependent gene expression changes.

**Fig. 6:**
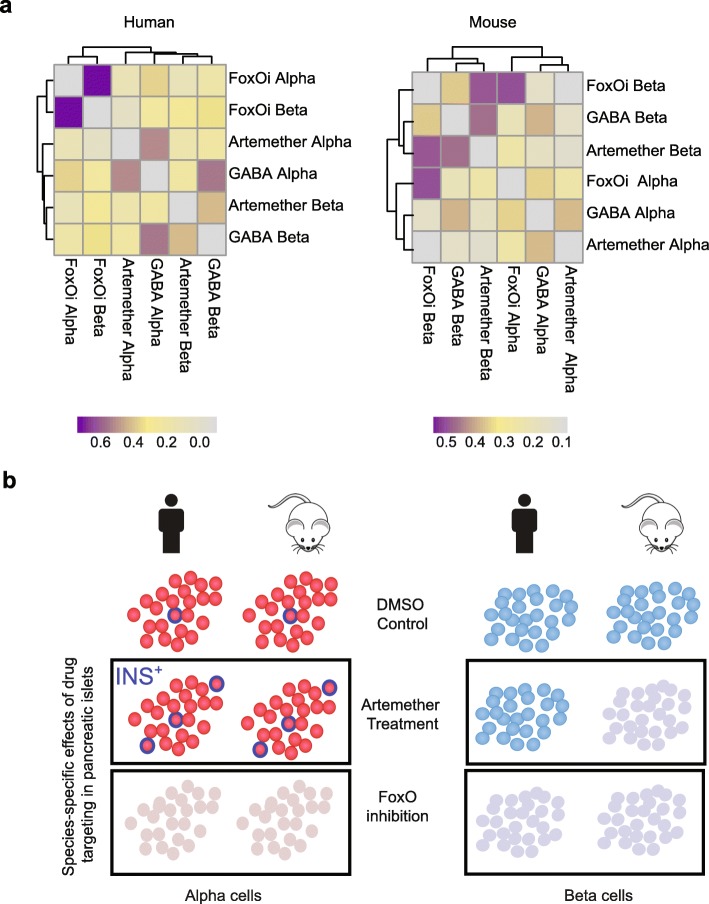
Artemether effects on beta cells are species dependent. **a** Spearman correlations of log fold changes of comparisons of 1 μM FoxOi, 10 μM artemether, or 100 μM GABA to DMSO for alpha and beta cells from mouse and human islets. **b** FoxOi induces dedifferentiation of alpha and beta cells in mouse and human, while artemether increases the fraction of INS^+^ alpha cells. In beta cells, artemether effects are species dependent; in mouse, the drug induces beta cell dedifferentiation, while in human there are no strong effects

Correlating the transcription changes between individual samples further supports the finding that these drugs exert different effects in mouse and human islet cells (Fig. 6).

## Discussion

We developed, validated, and applied a method to experimentally determine and computationally remove contaminating transcripts in single-cell RNA-seq data. We demonstrated that droplet-based single-cell transcriptomes can contain up to 20% contaminating transcripts, likely derived from cell-free RNA of lysing cells. This effect is most relevant for highly abundant transcripts, and it can result in incorrect conclusions.

Housekeeping genes with similar levels of expression across cell types tend to be among the most highly expressed genes, which is a likely reason why this type of contamination has not constituted a major limitation for primarily qualitative studies of cell type composition in primary tissues. In islet cells, however, the most highly expressed genes and most abundant contaminating transcripts are the cell-type-specific hormone genes, allowing us to more easily detect contamination and to develop a method to computationally remove it. While the extent of contamination is dependent on the tissue of origin and culture conditions, a baseline level of transcript redistribution is likely to occur in all droplet-based scRNA-seq samples. For this reason, we recommend including cross-species spike-in cells during the sample preparation workflow. Given the high number of single-cell transcriptomics studies currently ongoing, future standardization to a common extensively characterized set of spike-in cells to be used across a broad range of studies would be highly desirable to maximize data quality, particularly for future reference data sets.

To understand drug effects on islet cells, we analyzed data of a total of 107,192 islet single cells for cell-type-specific relative transcription changes caused by FoxOi, artemether, and GABA compared to DMSO. Previous work has shown that genetic inhibition of FOXO signaling in beta cells induces their dedifferentiation. Here we show that the effect can be phenocopied by a pharmacological inhibitor and is conserved in both mouse and human islets (Fig. 6b). We extend these findings to show that FoxOi also causes dedifferentation of alpha cells. FoxOi effects are correlated between alpha and beta cells, suggesting induction of a common signature possibly due to dedifferentiation (Fig. 6b). We did not observe strong effects of FoxOi on delta cells (Fig. S11), in line with the original publication [36] that describes such effects to occur specifically only after beta cell ablation. These data highlight the importance of intra-islet signaling for cellular transdifferentiation and validate FoxOi-mediated beta cell dedifferentiation as a model to identify inhibitors of the process.

In murine beta cells, transcription changes caused by GABA and artemether clustered with FoxOi, suggesting that these drugs also cause dedifferentiation as previously reported [8]. In human samples, however, these drugs formed a clearly separated cluster and did not cause downregulation of insulin (Fig. 6b). These data suggest that drug effects are species dependent; it will be important to understand what factors are responsible for the observed species differences.

Multiple in vivo data indicate that artemisinins are lowering blood glucose levels [37–42, 61], rather than causing diabetes by induction of beta cell dedifferentiation. Effects of the drug on body weight and other tissues including adipocytes and immune cells may contribute to altered glucose homeostasis, and it is currently unclear whether these effects are islet-independent as impairing alpha cell function can cause similar phenotypes [62, 63]. We originally observed impaired glucagon secretion and induction of insulin expression in alpha cells treated with artemether [36]. Two subsequent studies using lineage tracing mouse models failed to observe increases of alpha cell-derived beta cells following artemisinin treatment [8, 40]. Here we confirm direct effects of the drug on islet cells and show in both human and mouse islets that artemether induces expression of insulin and other beta cell-specific genes in a subpopulation of alpha cells. The levels of induction we observe after 3 day in vitro treatment are drastically lower than those present in beta cells, and different detection methods might contribute to the different conclusions of the studies. Overall, the gene expression changes caused by artemether and GABA appear correlated. While we did not observe the emergence of insulin-expressing alpha cells in samples from mouse islets treated with GABA, one human sample showed an increase from approximately 4 to 14% insulin-positive alpha cells.

Based on our and others’ studies, it is clear that artemisinin and GABA and FoxOi impact gene expression and cell identity in isolated pancreatic islets from mouse and human and that they alter glucose tolerance in mice in vivo. Our new spike-in-based method to obtain clean single-cell transcriptomes will aid future studies to clarify whether effects on islets cells are causative for the physiological effects on the whole organism level.

## Methods

### Donor information

**Table:** 

	Gender	Age (years)	BMI	HbA1c (%)	Provider	Institution
Donor I	Male	38	30.1	5.80	Tebu-bio	Prodo Laboratories
Donor II	Male	32	26.2	5.30	IIDP	University of Wisconsin
Donor III	Male	37	29.4	5.1	IIDP	The Scharp-Lacy Research Institute
Donor IV	Male	68	29.7	5.2	IIDP	The Scharp-Lacy Research Institute

### Mouse and human pancreatic islet sample preparation and single-cell RNA-seq

Human islets were obtained from the Integrated Islet Distribution Program (IIDP) and Tebu-bio. The current study was approved by the Ethics Committee of the Medical University of Vienna (EK-Nr. 1228/2015). Mouse islets were isolated in the Collombat laboratory according to standard protocols.

Human and mouse islets were cultured with 1 or 10 μM artemether (Cayman, Cat#11815) here referred as “Artemether” (stored at − 20 °C as 10 mM stock in DMSO), 10 μM artemether (Sigma-Aldrich, Cat#A9361) (stored at − 20 °C as 10 mM stock in DMSO) here referred as “A-Sigma” 100 μM GABA (Sigma-Aldrich, A2129) (stored at − 20 °C as 100 mM stock in water), 1 μM FoxOi (Sigma-Aldrich, Cat#AS1842856) (stored at − 20 °C as 1 mM stock in DMSO), and control DMSO (Sigma-Aldrich, Cat#41640) for 36 or 72 h in CMRL (Thermo-Fisher, Cat#11530037) medium supplemented with 10% fetal bovine serum (Thermo-Fisher, Cat#10500064), 5% penicillin/streptomycin (Thermo-Fisher, Cat#15140122), and 1 X Glutamax (Thermo-Fisher, Cat#35050038). Islets were maintained in culture at 37 °C in a 5% CO_2_ humidified atmosphere. After that period, islets were dissociated as follows: Islets were collected, centrifuged (1100 rpm, 3 min), and washed once with 10 ml of PBS. Islets were resuspended in 2 ml of Accutase (Sigma-Aldrich, Cat#A6964) and incubated at room temperature for 20 min with gentle intermittent mixing by pipetting. CMRL media with 10% FBS was used to stop the dissociation process. The dissociated islets were filtered through a 40-μm filter to obtain a single-cell suspension. A total of 20,000 cells were resuspended in 33 μl of PBS + 0.04% BSA. After the addition of 1 μl spike-in cells (equaling a total of 300–500 cells), the sample was run in the Chromium Single Cell Controller (10x Genomics) using version 2 chemistry for 3′ RNA sequencing as detailed in the manufacturer’s instructions. Individual libraries were diluted and pooled equimolarly, followed by sequencing on Illumina HiSeq 3000/4000 machines using the 75-bp paired-end setup.

### Preparation of spike-in cells

Human Jurkat cells and mouse 32D cells were cultured over 10 days and grown to a density of 10^6^ cells/ml. Cells were mixed in equal amounts and a total of 10^6^ mixed cells was pooled and centrifuged at 500*g* for 5 min at room temperature. The cells were washed once in PBS. After the last wash, the supernatant was reduced to 200 μl and the pellet was resuspended. A total of 800 μl of ice-cold pure methanol was added drop by drop to the cells along with constant mixing of the cell suspension. The cell-methanol mix was incorporated on ice for at least 15 min and transferred to − 80 °C for long-term storage. In preparation of each single-cell RNA-seq run, 100 μl of the methanol-fixed cells was supplemented with 900 μl ice-cold PBS slowly administered and the fully resuspended cells were centrifuged for 5 min at 4 °C at 3000*g*. The resulting pellet was resuspended in 100 μl of ice-cold PBS, and the cells were counted on a CASY cell counting system.

### Computational methods

#### Single-cell RNA-seq data preprocessing

Illumina BAM files were demultiplexed using cellranger *mkfastq* (version 2.1.0). Human samples were aligned to human and mouse to mouse reference genomes using cellranger *count*. Reference spike-in samples were aligned to both the mouse and human reference genomes. All samples were further aligned to the combined human/mouse reference genome (version 1.2.0). Samples were combined using cellranger *aggr* without down-sampling. Further data processing was carried out in R (3.4.0). Data were imported using the function *Read10X* from the Seurat package (version 2.0.1). Reads were transformed to transcripts per million (TPM) and log transformed (natural logarithm of TPM + 1) as done by Seurat. Barcodes with more than 500 UMIs and more than 200 genes were labeled as cells. In total, we obtained 35,333 (including 1420 spike-ins) mouse and 128,810 human (including 1778 spike-ins) single-cell transcriptomes.

#### Contamination factor and signature estimation from cross-species spike-ins

Cross-species spike-ins for each sample were identified by the ratio of reads aligned to human and mouse reference genomes, with cells with a log_2_ cutoff of 2 and − 2 used to label human and mouse cells, respectively. Ambiguous cells, which had reads from both human and mouse cells, were removed from the analysis. The contamination factor (*f*_*c*_) in cross-species spike-ins was estimated as the fraction of reads aligned to the sample genome divided by all aligned reads in the human/mouse combined alignment. Contamination signatures of each sample were obtained by dividing gene expression (TPM values) of cross-species spike-ins by (1 − *f*_c_) and subtracting the reference signature for each cell. These contamination values were normalized by setting negative values to zero, scaled up to reflect TPMs, and then averaged across cells for each sample, thus producing sample-level contamination signature estimates.

#### Contamination factor prediction and correction of expression values

To obtain contamination factors in cells where no experimental gold standard is available, we fitted models that predict contamination factor from individual islet marker gene expression (TPM values) in cross-species spike-ins using linear models without bias terms. Linear models were fitted using the *lm* function in R. The intersect of predefined islet marker genes with the top 100 contaminating genes was selected, which resulted in seven genes: INS, GCG, TTR, SST, PPY, IAPP, and REG1A for human and Ins1, Ins2, Gcg, Ttr, Iapp, Ppy, and Sst for mouse. Medians of predictions across genes were used as the final prediction. Predictions were capped at 0.2 in mouse and 0.1 in human. In addition, three-fold cross-validated estimates were used to evaluate generalizability of the predictions.

To correct expression values, contamination signatures were multiplied by *f*_*c*_ and subtracted from each cell’s expression values (TPM). Negative values were set to zero, and values were scaled up to reflect TPM.

The spike-in decontamination method was applied to all human and mouse samples in the dataset. After decontamination, we noticed that one human sample (GABA in donor II) captured a large portion of total cells (21,978 of 164,143 human cells) but had very low quality (Fig. S4F) and higher contamination after correction (Fig. 2c). Since we only expect roughly 10,000 cells from each sample, this case was deemed an outlier and excluded from further analysis, reducing the number of human cells to 106,832 (including 3529 spike-ins), which together with the 35,333 mouse cells (including 1420 spike-ins) resulted in a total number of 142,165 cells.

#### Cell type assignment

Clusters of cells were identified through principal component analysis, t-distributed stochastic neighborhood embedding (t-SNE), and clustering of the corrected data by the Seurat package (version 2.0.1). “Resolution” parameter of 0.5 was used in the cluster assignment. Same-species spike-ins were identified by correlating all cells to reference spike-ins, selecting cells with Pearson correlation greater than 0.9, and then assigning the cluster enriched for these cells as same-species spike-ins. Non-endocrine cells clustered individually and were assigned according to cluster marker genes.

Endocrine cells were not separated in the initial clustering. Clustering and t-SNE analysis was therefore repeated on endocrine cells separately for visualization. Clear representatives of alpha, beta, gamma, and delta cells were assigned using a log(TPM) threshold of 7.5 for insulin, glucagon, somatostatin, and pancreatic polypeptide. In addition, a cluster expressing high levels of REG1A was assigned as “Acinar like” in human samples. Then, a machine learning-based approach was used to assign cells to cell types: A elastic net regularized logistic regression classifier was trained to predict cell type. The cv.glmnet function of the glmnet package in R was used with “alpha” parameter set to 1, “family” set to “multinomial,” and “nfolds” set to 5. This model was trained to predict cell type for all cell types with greater than 50 representatives in DMSO samples in each organism. The four hormones used to assign cells in this procedure were removed. To reduce class imbalance, training data was subsampled to maximally 100 cells per cell type and sample. Cell type was then computationally predicted for all previously unassigned cells using the predict function. Clusters of non-endocrine cells, where there was no expression of marker genes or clear profile corresponding to a specific cell type, were labeled with numbers.

Finally, we used cell type predictions to also remove potential doublets. We based this filter on the rationale that singlets should have a high class probability for only one class whereas doublets have high class probability for more than one class [64, 65]. We thus calculated the ratio between the highest and second highest class probability for each cell, and removed all cells with lower than 3-fold difference as doublets. In addition, we implemented a second filter for doublets cells based on the number of genes per cell (*N*_genes_). For each cell type and sample, we identified the expected *N*_genes_ as the value of *N*_genes_ with the maximal density. Next, we removed all cells with *N*_genes_ greater than twice the expected *N*_genes_ of the respective cell type and sample.

#### Differential expression analysis

Differentially expressed genes were identified using the *FindMarkers* function of the Seurat package with parameters “negbinom” for “test.use”, 0.1 for “thresh.use.” Analysis was run twice: First for islet hormones INS, GCG, SST, and PPY individually. In order to control for potential biases of high expression of these hormones in cells, differential expression analysis for all other genes was done after removing these hormones and re-normalizing the data. *p* values were adjusted for multiple testing using *p.adjust* in R with method “BH.” Genes with ≥ ± 1.25-fold gene expression changes and adjusted *p* value smaller than 0.05 were kept as significant.

To compare log fold changes between conditions and between species, gene names were mapped using the HomoloGene database (build 68), where only one-to-one mappings of gene names between human and mouse were used (genes with one-to-many mappings were excluded). Genes with significant differential expression in any condition were used, and Spearman correlation was calculated to assess similarity of changes.

#### Correlation to beta and alpha cell signatures

Marker genes of alpha and beta cells were obtained by comparing the cell belonging to either type to all other cells using the *FindMarkers* function of the Seurat package with parameters “roc” for “test.use”, 0.7 for “thresh.use.” Genes with receiver operating characteristic (ROC) greater than 0.75 were used as marker genes. Average alpha cell and beta cell expression profiles were generated by taking the mean log(TPM) values of each gene across all cells of each type from DMSO-treated controls. Individual cells were then correlated to average profiles using the Spearman correlation, across all genes identified as marker genes of either alpha or beta cells.

### Availability of data and materials

10 X next-generation sequencing data are available in the NCBI GEO, under accession number GSE147203 [66]. Drop-seq next-generation sequencing data are available in the NCBI GEO, under accession number GSE147202 [67]. The authors declare that all other data supporting the findings of this study are within the manuscript and its supplementary files.

The computational pipeline to analyze this dataset is open source and publicly available at https://github.com/epigen/Artemether_scRNA [68].

## Supplementary information

**Additional file 1.** Supplementary Figures S1-S11 and corresponding legends

**Additional file 2: Table S1.** Significance values for correlation of corrected and raw data to reference spike-ins as shown in Fig. 2C, Fig. 2D and Additional file 1: Figure S3B.

**Additional file 3: Table S2.** Cell numbers and filtering workflow as shown in Additional file 1: Figure S6.

**Additional file 4: Table S3.** Differential gene expression in all cell types and treatments in mouse and human as shown in Figs. 4A,B,C, Fig. 5A, D, E and Fig. 6A.

**Additional file 5: Table S4.** Significance values for insulin expression in beta cells as shown in Fig. 4A and Fig. 5C.

**Additional file 6: Table S5.** GSEA for significant genes changed in alpha and beta cells treated with artemether and FoxOi.

**Additional file 7: Table S6.** Significance values for proportion of alpha cells with insulin expression as shown in Fig. 4A.

**Additional file 8: Table S7.** Differential gene expression in alpha insulin+ cells as shown in Fig. 5B.

**Additional file 9: Table S8.** GSEA for significant genes changed in alpha insulin+ cells.

**Additional file 10.** Review history.

## References

[1] Schaum N, Karkanias J, Neff NF, May AP, Quake SR, Wyss-Coray T, Darmanis S, Batson J, Botvinnik O, Chen MB (2018). Single-cell transcriptomics of 20 mouse organs creates a Tabula Muris. Nature.

[2] Han X, Wang R, Zhou Y, Fei L, Sun H, Lai S, Saadatpour A, Zhou Z, Chen H, Ye F (2018). Mapping the mouse cell atlas by microwell-seq. Cell.

[3] Regev A, Teichmann SA, Lander ES, Amit I, Benoist C, Birney E, Bodenmiller B, Campbell P, Carninci P, Clatworthy M, et al. The human cell atlas. Elife. 2017;6. 10.7554/eLife.27041 PMC5762154 29206104

[4] Tabula Muris Consortium (2018). Single-cell transcriptomics of 20 mouse organs creates a Tabula Muris. Nature.

[5] Gutierrez GD, Bender AS, Cirulli V, Mastracci TL, Kelly SM, Tsirigos A, Kaestner KH, Sussel L (2017). Pancreatic beta cell identity requires continual repression of non-beta cell programs. J Clin Invest.

[6] Butler AE, Campbell-Thompson M, Gurlo T, Dawson DW, Atkinson M, Butler PC (2013). Marked expansion of exocrine and endocrine pancreas with incretin therapy in humans with increased exocrine pancreas dysplasia and the potential for glucagon-producing neuroendocrine tumors. Diabetes.

[7] Thorel F, Nepote V, Avril I, Kohno K, Desgraz R, Chera S, Herrera PL (2010). Conversion of adult pancreatic alpha-cells to beta-cells after extreme beta-cell loss. Nature.

[8] van der Meulen T, Lee S, Noordeloos E, Donaldson CJ, Adams MW, Noguchi GM, Mawla AM, Huising MO (2018). Artemether does not turn alpha cells into beta cells. Cell Metab.

[9] Enge M, Arda HE, Mignardi M, Beausang J, Bottino R, Kim SK, Quake SR (2017). Single-cell analysis of human pancreas reveals transcriptional signatures of aging and somatic mutation patterns. Cell.

[10] Teo AKK, Lim CS, Cheow LF, Kin T, Shapiro JA, Kang NY, Burkholder W, Lau HH (2018). Single-cell analyses of human islet cells reveal de-differentiation signatures. Cell Death Discov.

[11] Goldstein LD, Chen YJ, Dunne J, Mir A, Hubschle H, Guillory J, Yuan W, Zhang J, Stinson J, Jaiswal B (2017). Massively parallel nanowell-based single-cell gene expression profiling. BMC Genomics.

[12] Qiu WL, Zhang YW, Feng Y, Li LC, Yang L, Xu CR (2017). Deciphering pancreatic islet beta cell and alpha cell maturation pathways and characteristic features at the single-cell level. Cell Metab.

[13] Zeng C, Mulas F, Sui Y, Guan T, Miller N, Tan Y, Liu F, Jin W, Carrano AC, Huising MO (2017). Pseudotemporal ordering of single cells reveals metabolic control of postnatal beta cell proliferation. Cell Metab.

[14] Muraro MJ, Dharmadhikari G, Grun D, Groen N, Dielen T, Jansen E, van Gurp L, Engelse MA, Carlotti F, de Koning EJ, van Oudenaarden A (2016). A single-cell transcriptome atlas of the human pancreas. Cell Syst.

[15] Segerstolpe A, Palasantza A, Eliasson P, Andersson EM, Andreasson AC, Sun X, Picelli S, Sabirsh A, Clausen M, Bjursell MK (2016). Single-cell transcriptome profiling of human pancreatic islets in health and type 2 diabetes. Cell Metab.

[16] Xin Y, Kim J, Okamoto H, Ni M, Wei Y, Adler C, Murphy AJ, Yancopoulos GD, Lin C, Gromada J (2016). RNA sequencing of single human islet cells reveals type 2 diabetes genes. Cell Metab.

[17] Xin Y, Okamoto H, Kim J, Ni M, Adler C, Cavino K, Na E, Murphy AJ, Yancopoulos GD, Lin C, Gromada J (2016). Single-cell RNAseq reveals that pancreatic beta-cells from very old male mice have a young gene signature. Endocrinology.

[18] Wang YJ, Schug J, Won KJ, Liu C, Naji A, Avrahami D, Golson ML, Kaestner KH (2016). Single-cell transcriptomics of the human endocrine pancreas. Diabetes.

[19] Li J, Klughammer J, Farlik M, Penz T, Spittler A, Barbieux C, Berishvili E, Bock C, Kubicek S (2016). Single-cell transcriptomes reveal characteristic features of human pancreatic islet cell types. EMBO Rep.

[20] Dorajoo R, Ali Y, Tay VSY, Kang J, Samydurai S, Liu J, Boehm BO (2017). Single-cell transcriptomics of East-Asian pancreatic islets cells. Sci Rep.

[21] Bengtsson M, Stahlberg A, Rorsman P, Kubista M (2005). Gene expression profiling in single cells from the pancreatic islets of Langerhans reveals lognormal distribution of mRNA levels. Genome Res.

[22] Xin Y, Dominguez Gutierrez G, Okamoto H, Kim J, Lee AH, Adler C, Ni M, Yancopoulos GD, Murphy AJ, Gromada J (2018). Pseudotime ordering of single human beta-cells reveals states of insulin production and unfolded protein response. Diabetes.

[23] Baron M, Veres A, Wolock SL, Faust AL, Gaujoux R, Vetere A, Ryu JH, Wagner BK, Shen-Orr SS, Klein AM (2016). A single-cell transcriptomic map of the human and mouse pancreas reveals inter- and intra-cell population structure. Cell Syst.

[24] Blodgett DM, Nowosielska A, Afik S, Pechhold S, Cura AJ, Kennedy NJ, Kim S, Kucukural A, Davis RJ, Kent SC (2015). Novel observations from next-generation RNA sequencing of highly purified human adult and fetal islet cell subsets. Diabetes.

[25] Katsuta H, Akashi T, Katsuta R, Nagaya M, Kim D, Arinobu Y, Hara M, Bonner-Weir S, Sharma AJ, Akashi K, Weir GC (2010). Single pancreatic beta cells co-express multiple islet hormone genes in mice. Diabetologia.

[26] Xin Y, Kim J, Ni M, Wei Y, Okamoto H, Lee J, Adler C, Cavino K, Murphy AJ, Yancopoulos GD (2016). Use of the Fluidigm C1 platform for RNA sequencing of single mouse pancreatic islet cells. Proc Natl Acad Sci U S A.

[27] Chakravarthy H, Gu X, Enge M, Dai X, Wang Y, Damond N, Downie C, Liu K, Wang J, Xing Y (2017). Converting adult pancreatic islet alpha cells into beta cells by targeting both Dnmt1 and Arx. Cell Metab.

[28] Courtney M, Gjernes E, Druelle N, Ravaud C, Vieira A, Ben-Othman N, Pfeifer A, Avolio F, Leuckx G, Lacas-Gervais S (2013). The inactivation of Arx in pancreatic alpha-cells triggers their neogenesis and conversion into functional beta-like cells. PLoS Genet.

[29] Yang YP, Thorel F, Boyer DF, Herrera PL, Wright CV (2011). Context-specific alpha- to-beta-cell reprogramming by forced Pdx1 expression. Genes Dev.

[30] Collombat P, Xu X, Ravassard P, Sosa-Pineda B, Dussaud S, Billestrup N, Madsen OD, Serup P, Heimberg H, Mansouri A (2009). The ectopic expression of Pax4 in the mouse pancreas converts progenitor cells into alpha and subsequently beta cells. Cell.

[31] Bramswig NC, Everett LJ, Schug J, Dorrell C, Liu C, Luo Y, Streeter PR, Naji A, Grompe M, Kaestner KH (2013). Epigenomic plasticity enables human pancreatic alpha to beta cell reprogramming. J Clin Invest.

[32] Dhawan S, Georgia S, Tschen SI, Fan G, Bhushan A (2011). Pancreatic beta cell identity is maintained by DNA methylation-mediated repression of Arx. Dev Cell.

[33] Talchai C, Xuan S, Lin HV, Sussel L, Accili D (2012). Pancreatic beta cell dedifferentiation as a mechanism of diabetic beta cell failure. Cell.

[34] Furuyama K, Chera S, van Gurp L, Oropeza D, Ghila L, Damond N, Vethe H, Paulo JA, Joosten AM, Berney T (2019). Diabetes relief in mice by glucose-sensing insulin-secreting human alpha-cells. Nature.

[35] Nagashima T, Shigematsu N, Maruki R, Urano Y, Tanaka H, Shimaya A, Shimokawa T, Shibasaki M (2010). Discovery of novel forkhead box O1 inhibitors for treating type 2 diabetes: improvement of fasting glycemia in diabetic db/db mice. Mol Pharmacol.

[36] Chera S, Baronnier D, Ghila L, Cigliola V, Jensen JN, Gu G, Furuyama K, Thorel F, Gribble FM, Reimann F, Herrera PL (2014). Diabetes recovery by age-dependent conversion of pancreatic delta-cells into insulin producers. Nature.

[37] Li J, Casteels T, Frogne T, Ingvorsen C, Honore C, Courtney M, Huber KVM, Schmitner N, Kimmel RA, Romanov RA (2017). Artemisinins target GABAA receptor signaling and impair alpha cell identity. Cell.

[38] Guo Y, Fu W, Xin Y, Bai J, Peng H, Fu L, Liu J, Li L, Ma Y, Jiang H (2018). Antidiabetic and antiobesity effects of artemether in db/db mice. Biomed Res Int.

[39] Xiang M, Chen Z, He L, Xiong G, Lu J (2019). Transcription profiling of artemisinin-treated diabetic nephropathy rats using high-throughput sequencing. Life Sci.

[40] Li Z, Shi X, Liu J, Shao F, Huang G, Zhou Z, Zheng P. Artesunate prevents type 1 diabetes in NOD mice mainly by inducing protective IL-4-producing T cells and regulatory T cells. FASEB J. 2019:fj201900146R. 10.1096/fj.201900146R 30916998

[41] Ackermann AM, Moss NG, Kaestner KH. GABA and artesunate do not induce pancreatic alpha-to-beta cell transdifferentiation in vivo. Cell Metab. 2018;28:787–92 e783. 10.1016/j.cmet.2018.07.002 PMC8129910 30057067

[42] Han P, Wang Y, Zhan H, Weng W, Yu X, Ge N, Wang W, Song G, Yi T, Li S (2019). Artemether ameliorates type 2 diabetic kidney disease by increasing mitochondrial pyruvate carrier content in db/db mice. Am J Transl Res.

[43] Tian J, Dang H, Middleton B, Kaufman DL (2017). Clinically applicable GABA receptor positive allosteric modulators promote ss-cell replication. Sci Rep.

[44] Untereiner A, Abdo S, Bhattacharjee A, Gohil H, Pourasgari F, Ibeh N, Lai M, Batchuluun B, Wong A, Khuu N (2019). GABA promotes beta-cell proliferation, but does not overcome impaired glucose homeostasis associated with diet-induced obesity. FASEB J.

[45] Purwana I, Zheng J, Li X, Deurloo M, Son DO, Zhang Z, Liang C, Shen E, Tadkase A, Feng ZP (2014). GABA promotes human beta-cell proliferation and modulates glucose homeostasis. Diabetes.

[46] Tian J, Dang H, Karashchuk N, Xu I, Kaufman DL (2019). A clinically applicable positive allosteric modulator of GABA receptors promotes human beta-cell replication and survival as well as GABA’s ability to inhibit inflammatory T cells. J Diabetes Res.

[47] Tian J, Dang H, Nguyen AV, Chen Z, Kaufman DL (2014). Combined therapy with GABA and proinsulin/alum acts synergistically to restore long-term normoglycemia by modulating T-cell autoimmunity and promoting beta-cell replication in newly diabetic NOD mice. Diabetes.

[48] Tian J, Dang H, Chen Z, Guan A, Jin Y, Atkinson MA, Kaufman DL (2013). gamma-Aminobutyric acid regulates both the survival and replication of human beta-cells. Diabetes.

[49] Son DO, Liu W, Li X, Prud'homme GJ, Wang Q (2019). Combined effect of GABA and glucagon-like peptide-1 receptor agonist on cytokine-induced apoptosis in pancreatic beta-cell line and isolated human islets. J Diabetes.

[50] Li J, Zhang Z, Liu X, Wang Y, Mao F, Mao J, Lu X, Jiang D, Wan Y, Lv JY (2015). Study of GABA in healthy volunteers: pharmacokinetics and pharmacodynamics. Front Pharmacol.

[51] Feng AL, Xiang YY, Gui L, Kaltsidis G, Feng Q, Lu WY (2017). Paracrine GABA and insulin regulate pancreatic alpha cell proliferation in a mouse model of type 1 diabetes. Diabetologia.

[52] Soltani N, Qiu H, Aleksic M, Glinka Y, Zhao F, Liu R, Li Y, Zhang N, Chakrabarti R, Ng T (2011). GABA exerts protective and regenerative effects on islet beta cells and reverses diabetes. Proc Natl Acad Sci U S A.

[53] Hardwick SA, Deveson IW, Mercer TR (2017). Reference standards for next-generation sequencing. Nat Rev Genet.

[54] Griffiths JA, Richard AC, Bach K, Lun ATL, Marioni JC (2018). Detection and removal of barcode swapping in single-cell RNA-seq data. Nat Commun.

[55] Young MD, Behjati S. SoupX removes ambient RNA contamination from droplet based single cell RNA sequencing data. bioRxiv. 2018. preprint at 10.1101/303727. 10.1093/gigascience/giaa151 PMC7763177 33367645

[56] Kim-Muller JY, Kim YJ, Fan J, Zhao S, Banks AS, Prentki M, Accili D (2016). FoxO1 deacetylation decreases fatty acid oxidation in beta-cells and sustains insulin secretion in diabetes. J Biol Chem.

[57] Kim-Muller JY, Fan J, Kim YJ, Lee SA, Ishida E, Blaner WS, Accili D (2016). Aldehyde dehydrogenase 1a3 defines a subset of failing pancreatic beta cells in diabetic mice. Nat Commun.

[58] Fabregat A, Jupe S, Matthews L, Sidiropoulos K, Gillespie M, Garapati P, Haw R, Jassal B, Korninger F, May B (2018). The reactome pathway knowledgebase. Nucleic Acids Res.

[59] Huang R, Grishagin I, Wang Y, Zhao T, Greene J, Obenauer JC, Ngan D, Nguyen D-T, Guha R, Jadhav A, et al. The NCATS BioPlanet – an integrated platform for exploring the universe of cellular signaling pathways for toxicology, systems biology, and chemical genomics. Front Pharmacol. 2019;10:445. 10.3389/fphar.2019.00445 PMC6524730 31133849

[60] Ackermann AM, Wang Z, Schug J, Naji A, Kaestner KH (2016). Integration of ATAC-seq and RNA-seq identifies human alpha cell and beta cell signature genes. Mol Metab.

[61] Lu P, Zhang FC, Qian SW, Li X, Cui ZM, Dang YJ, Tang QQ (2016). Artemisinin derivatives prevent obesity by inducing browning of WAT and enhancing BAT function. Cell Res.

[62] Furuta M, Yano H, Zhou A, Rouille Y, Holst JJ, Carroll R, Ravazzola M, Orci L, Furuta H, Steiner DF (1997). Defective prohormone processing and altered pancreatic islet morphology in mice lacking active SPC2. Proc Natl Acad Sci U S A.

[63] Anini Y, Mayne J, Gagnon J, Sherbafi J, Chen A, Kaefer N, Chretien M, Mbikay M (2010). Genetic deficiency for proprotein convertase subtilisin/kexin type 2 in mice is associated with decreased adiposity and protection from dietary fat-induced body weight gain. Int J Obes.

[64] DePasquale EAK, Schnell DJ, Van Camp PJ, Valiente-Alandi I, Blaxall BC, Grimes HL, Singh H, Salomonis N (2019). DoubletDecon: deconvoluting doublets from single-cell RNA-sequencing data. Cell Rep.

[65] Wolock SL, Lopez R, Klein AM (2019). Scrublet: computational identification of cell doublets in single-cell transcriptomic data. Cell Syst.

[66] Marquina-Sanchez B, Fortelny N, Farlik M, Vieira A, Collombat P, Bock C, Kubicek S. Single-cell RNA-seq with spike-in cells enables accurate quantification of cell-specific drug effects in pancreatic islets. All 10X next generation single-cell sequencing libraries presented in this publication. Gene Expression Omnibus. https://www.ncbi.nlm.nih.gov/geo/query/acc.cgi?acc=GSE147203. 2020. 10.1186/s13059-020-02006-2 PMC7201533 32375897

[67] Marquina-Sanchez B, Fortelny N, Farlik M, Vieira A, Collombat P, Bock C, Kubicek S. Single-cell RNA-seq with spike-in cells enables accurate quantification of cell-specific drug effects in pancreatic islets. All Drop-Seq next generation single-cell sequencing libraries presented in this publication. Gene Expression Omnibus. https://www.ncbi.nlm.nih.gov/geo/query/acc.cgi?acc=GSE147202. 2020. 10.1186/s13059-020-02006-2 PMC7201533 32375897

[68] Marquina-Sanchez B, Fortelny N, Farlik M, Vieira A, Collombat P, Bock C, Kubicek S. Single-cell RNA-seq with spike-in cells enables accurate quantification of cell-specific drug effects in pancreatic islets. computational pipeline. Github repository https://github.com/epigen/Artemether_scRNA. 2020. 10.1186/s13059-020-02006-2 PMC7201533 32375897

