# Supplementary material for: Single-cell RNA-seq with spike-in cells enables accurate quantification of cell-specific drug effects in pancreatic islets
Source: Genome Biol. 2020 May 6;21:106. doi: 10.1186/s13059-020-02006-2 (PMC7201533; doi:10.1186/s13059-020-02006-2)
Supplement: Supplementary file 1 — Additional file 1. Supplementary Figures S1-S11 and corresponding legends [file 13059_2020_2006_MOESM1_ESM.pdf]

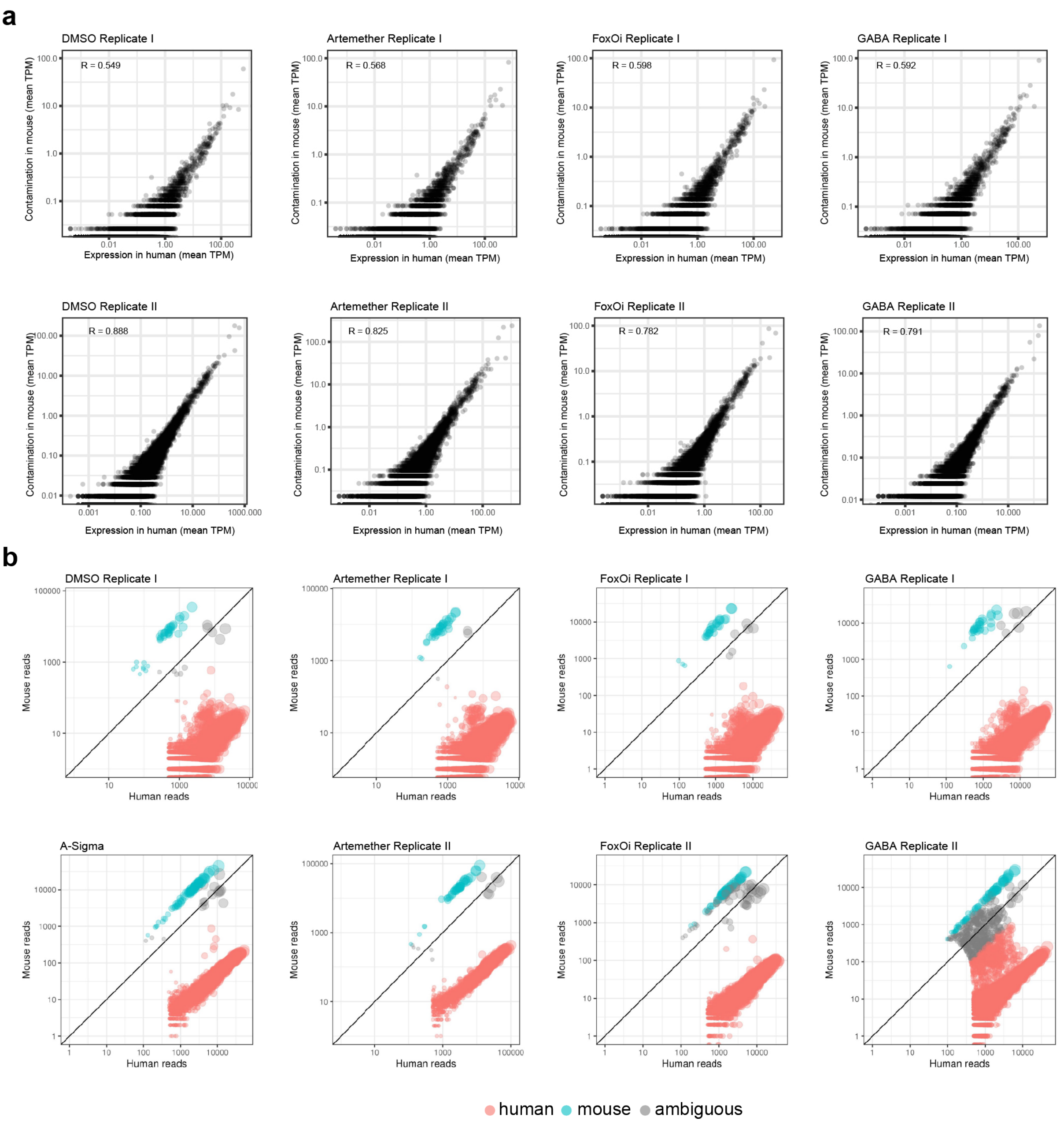

Figure S1: Cross-species spike-ins. (A) Average contamination in mouse spike-ins (y-axis) versus average expression in sample (human pancreatic cells, x-axis). Spearman correlation is shown in the plot. **(B)** Cross-species alignment of cells.

a

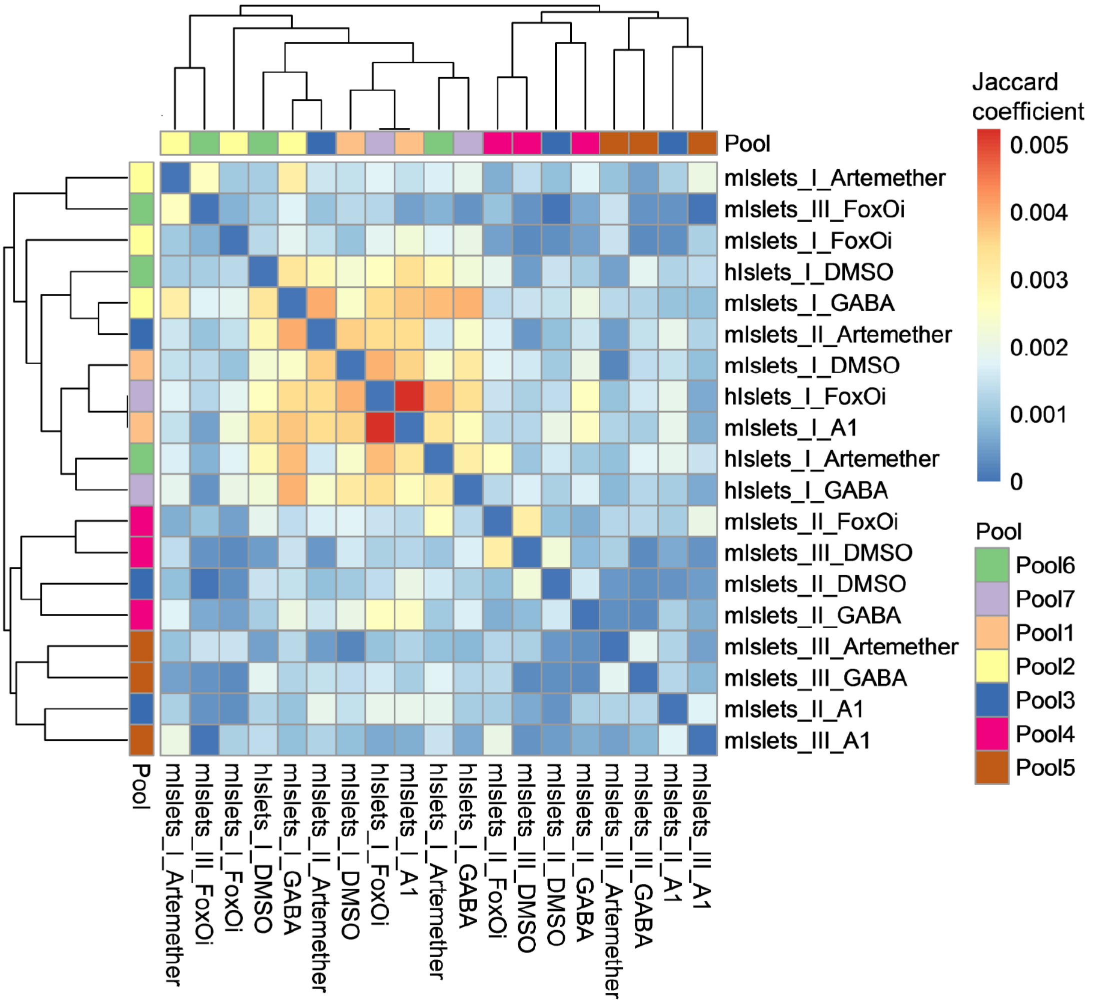

Figure S2: Contamination is not caused by index switching. Overlap in barcodes labeled as cells indicating the sequencing pool.

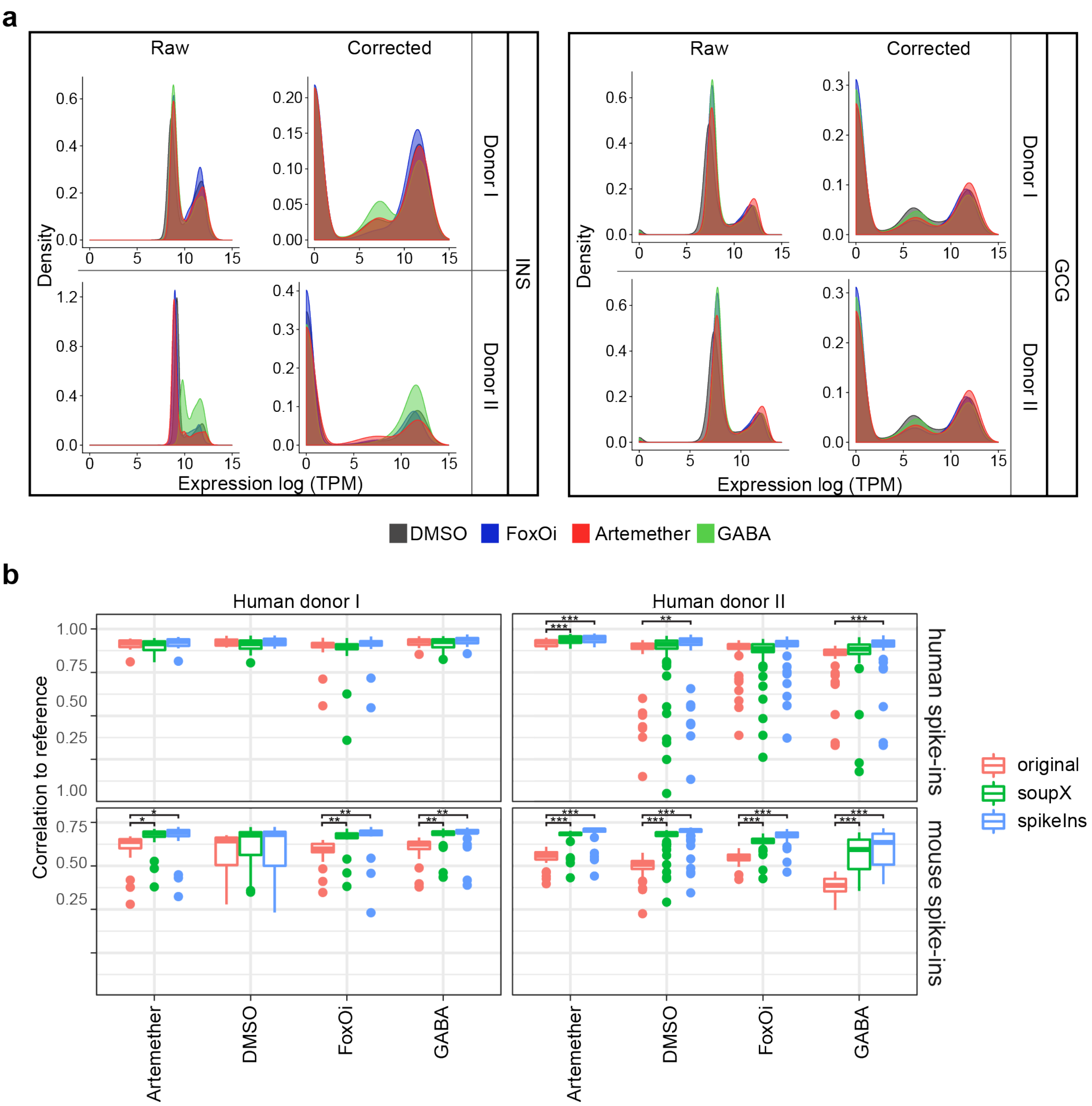

Figure S3: Data correction. (A) Raw and corrected insulin and glucagon expression in different human samples. **(B)** Pearson correlation to external reference spike-in transcriptomes of raw (red) and SoupX (green) or spike-in (blue) corrected gene expression values of the spike-ins cells.

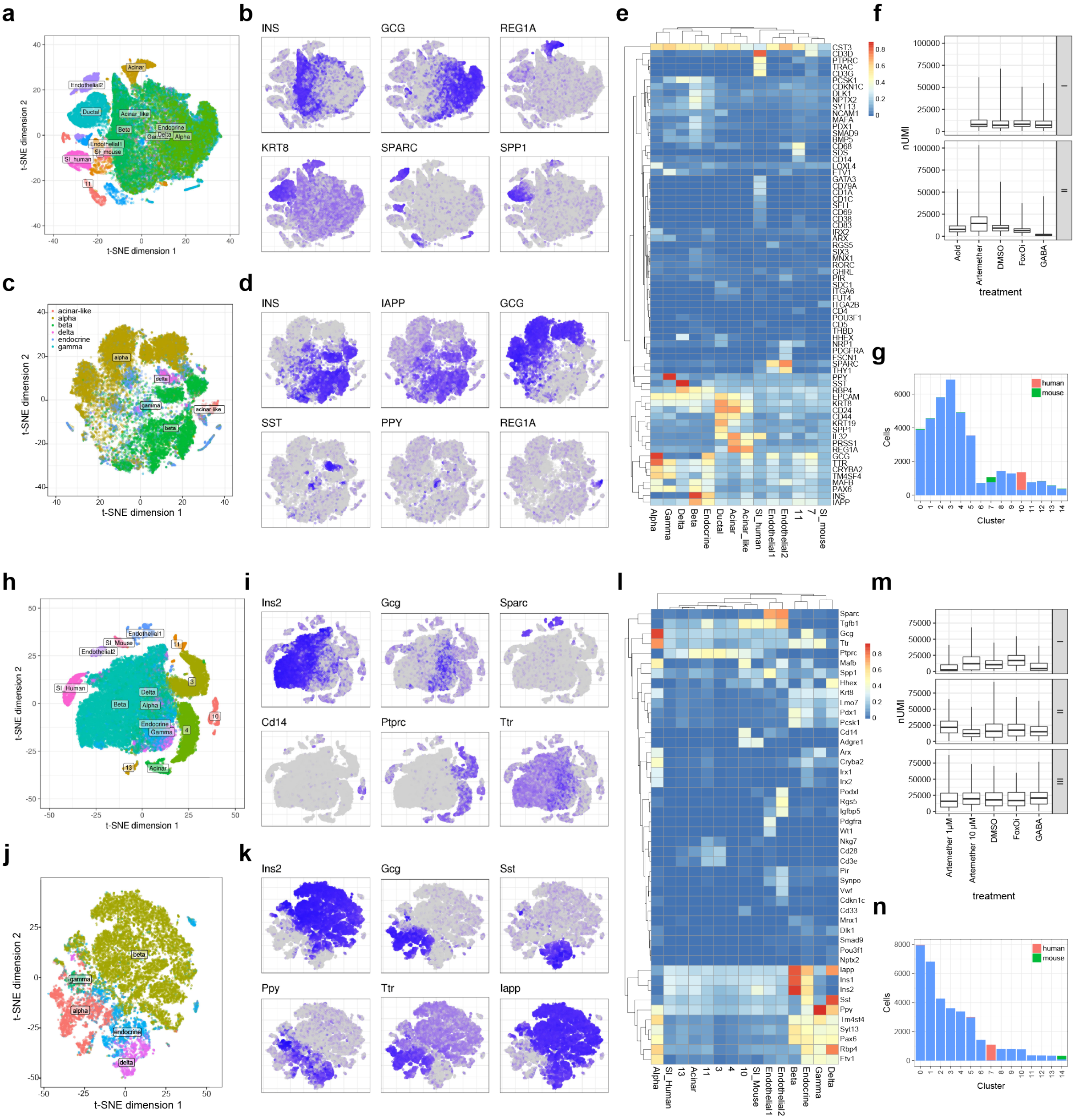

Figure S4: Cell type assignment. (A-G) Data from human islets. **(H-M)** Data from mouse islets. **(A,H)** t-SNE of all cells with **(B,I)** marker gene expression. **(C,J)** t-SNE of endocrine cells with **(D,K)** marker gene expression. **(E,L)** Average expression of marker genes in all clusters. No specific cell type could be assigned to the cell clusters labeled with numbers. **(F,M)** Number of unique molecular identifiers (UMIs) of all cells. **(G,N)** Enrichment for spike-ins in specific clusters.

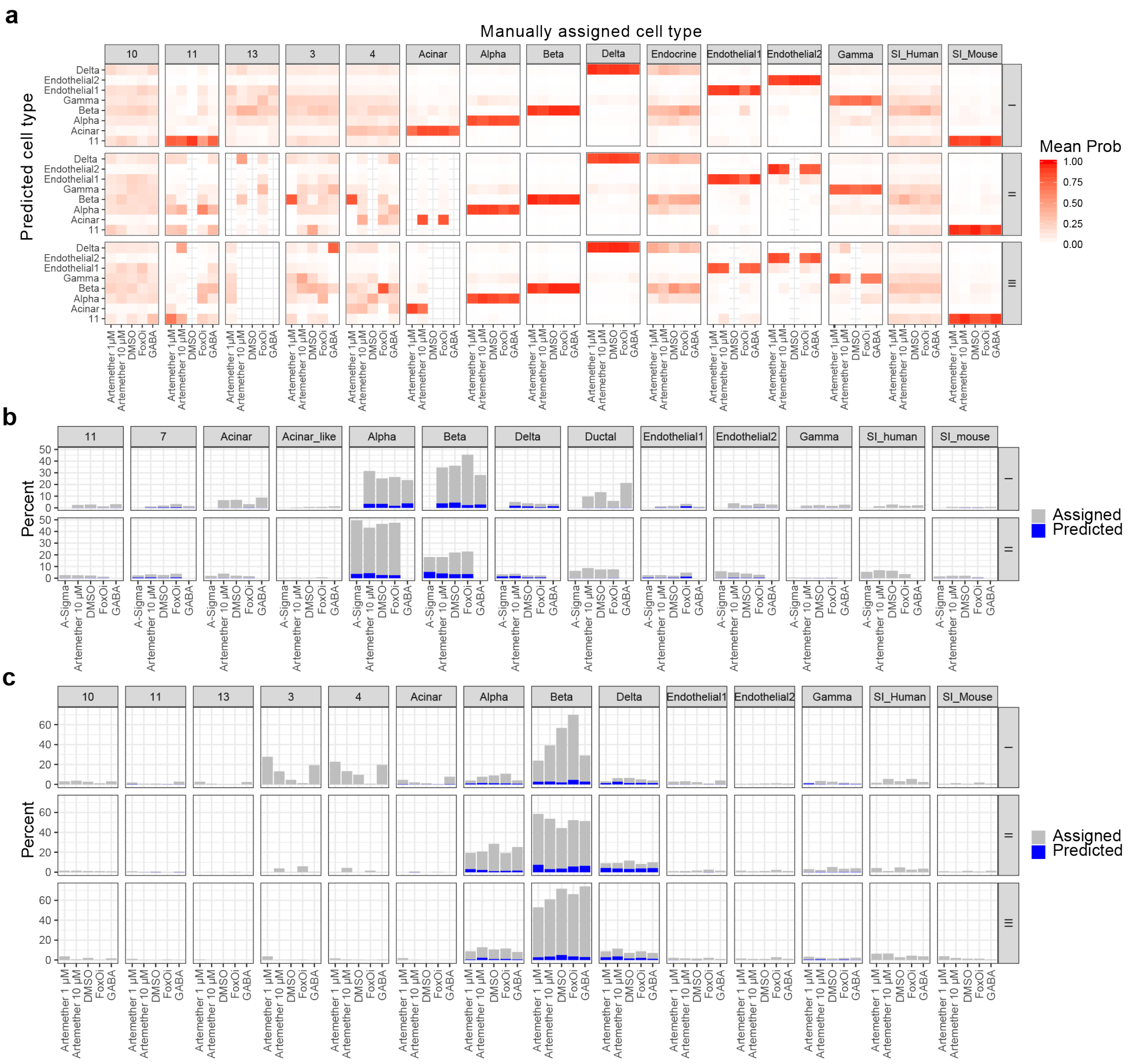

Figure S5: Cell type predictions. (A) Cell type probability predicted for each cell not used in training in mouse samples. Cell type reassignment based on predictions was only done for those cells not previously assignable, here labeled as 'Endocrine'. **(B,C)** Number of cells of each cell type in human **(B)** and mouse **(C)** islets. Number of computationally predicted cells shown in blue.

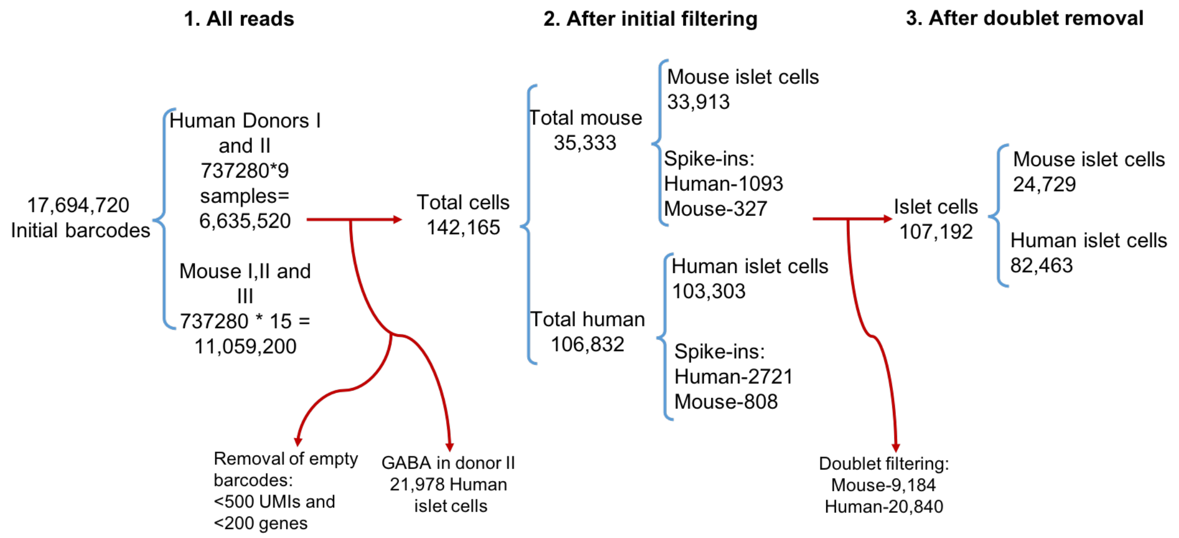

Figure S6: Overview of bioinformatic filtering and categorization workflow. First reads were transformed to transcripts per million. All barcodes with more than 500 UMIs and more than 200 genes were labelled as cells. Cells from human donor II treated with GABA were filtered out and doublets were removed based on the number of genes and cell type predictions.

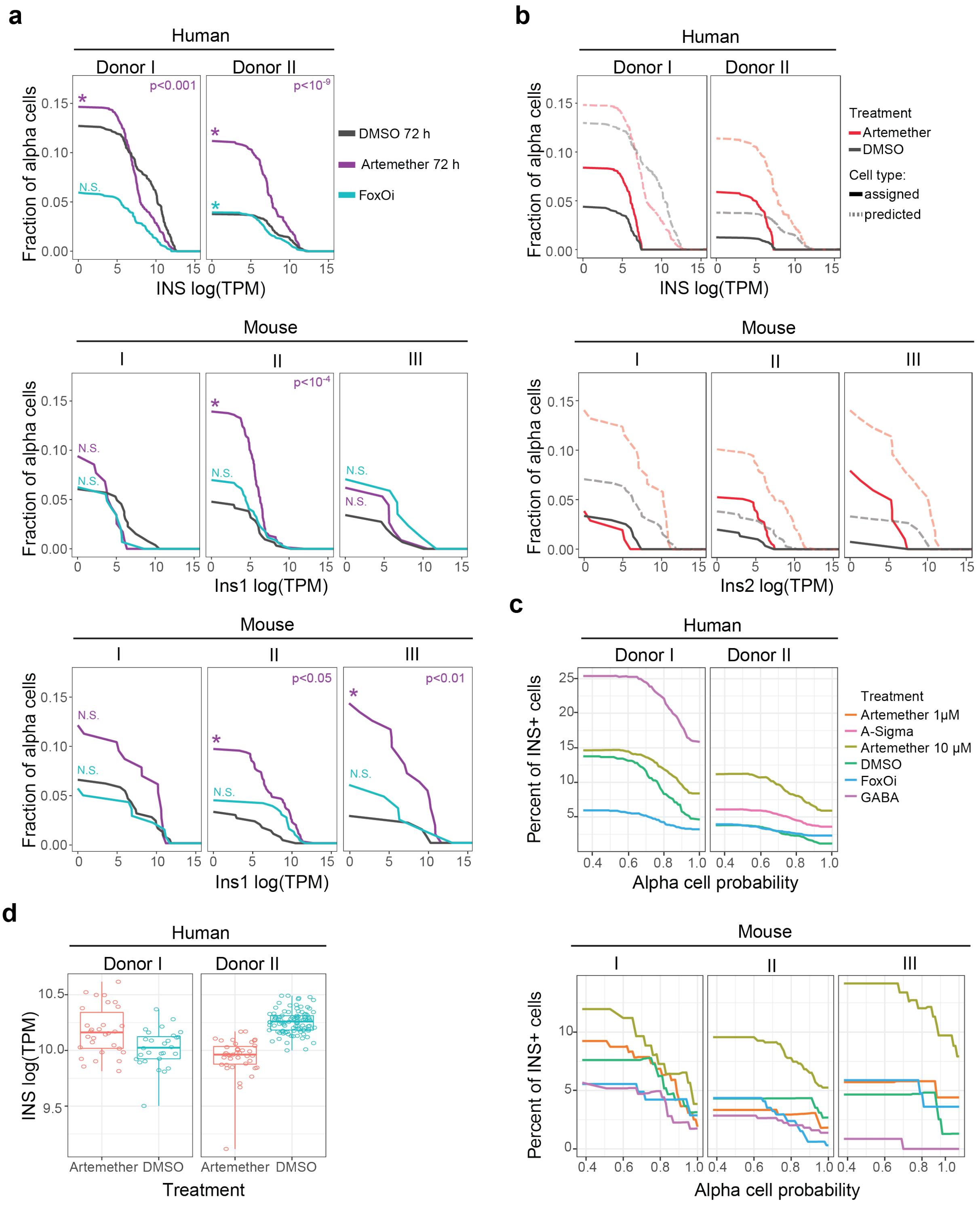

Figure S7: Insulin expression in alpha cells increases with artemether treatment and is variable among replicates. (A) Inverse cumulative distribution of insulin expression in assigned alpha cells from human (n=2) and mouse (n=3) islets treated with 10 μM FoxOi or 10 μM artemether or DMSO for 72 h. **(B)** Inverse cumulative distribution of insulin expression in assigned and predicted alpha cells from these human and mouse islets. **(C)** Percentage of INS+ alpha cells related to the alpha cell probability from the predictor in human and mouse islets. Assigned alpha cells are indicated as probability 1. **(D)** Raw insulin expression prior to spike-in correction in human islets.

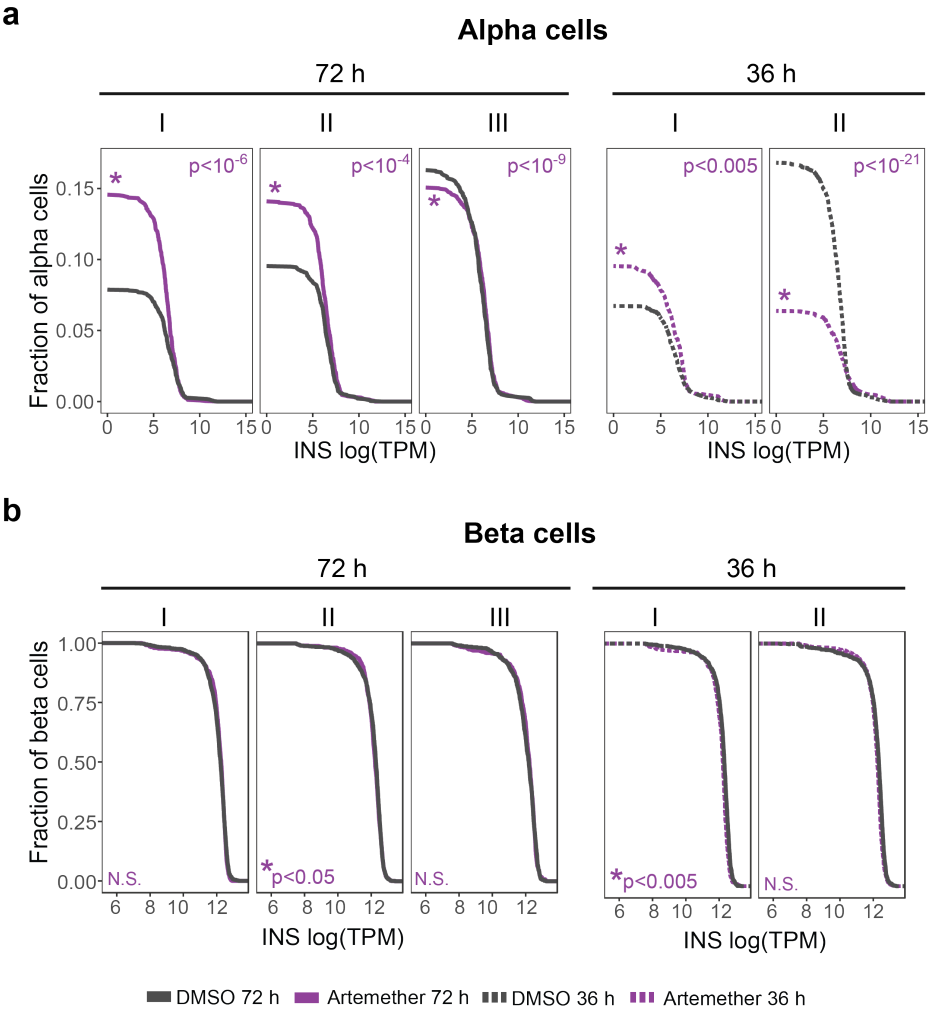

Figure S8: Artemether increases the fraction of alpha cells that express insulin in islets from a third human donor. (A) Inverse cumulative distribution of insulin expression from three replicates of islets from human donor III treated with 10 μM artemether or DMSO by duplicate for 36 h and by triplicate for 72 h in assigned alpha cells and **(B**) in assigned beta cells.

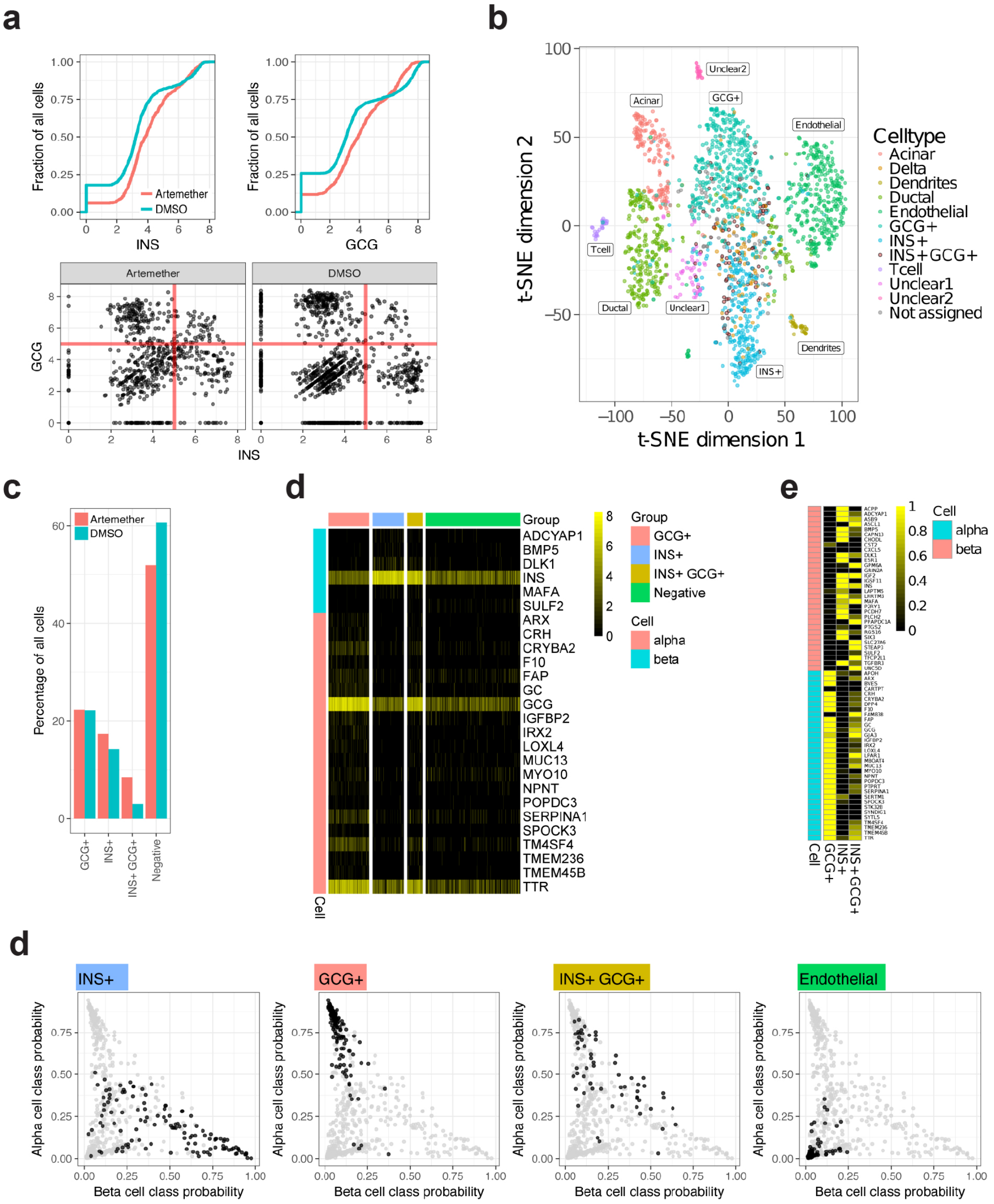

Figure S9: Drop-seq analysis of islets from human donor IV treated with artemether show similar results to 10X. (**A**) Insulin (INS) and glucagon (GCG) expression. Top: Cumulative distribution of glucagon (top right) and insulin (top left) expression in DMSO and artemether treated samples. Bottom: Insulin (x-axis) and Glucagon (y-axis) expression distinguishes alpha (INS$^-$ GCG$^+$), beta (INS$^+$ GCG$^-$), double positive (INS$^+$ GCG$^+$), and negative (INS$^-$ GCG$^-$) cells in all treatments. Red lines are the thresholds used to define cells (cutoff of 5 for both INS and GCG). (**B**) t-SNE plot of all cells. Cells were colored by the assigned cell type. (**C**) Percentage of cells in each group from all cells. INS$^+$ GCG$^+$ cells are enriched 3.0-fold in artemether compared to DMSO ($p < 10^{-6}$, Fisher Exact test). (**D**) Expression of alpha and beta cell marker genes (extracted from [65]) in artemether treated cells. Only genes with reads in more than 20 cells are shown. (**E**) Normalized mean expression of all marker genes (from [60]) in artemether treated cells. Mean gene expression of all cells in each group is shown and was normalized to a minimum of 0 and maximum of 1 for each gene. (**F**) Beta cell and alpha cell class probabilities (determined without INS, GCG, and SST expression) of artemether treated cells separated by cell type. (**G**) Delta cell class probabilities of INS$^+$, GCG$^+$, INS$^+$ GCG$^+$, and Endothelial cells plotted as cumulative distribution.

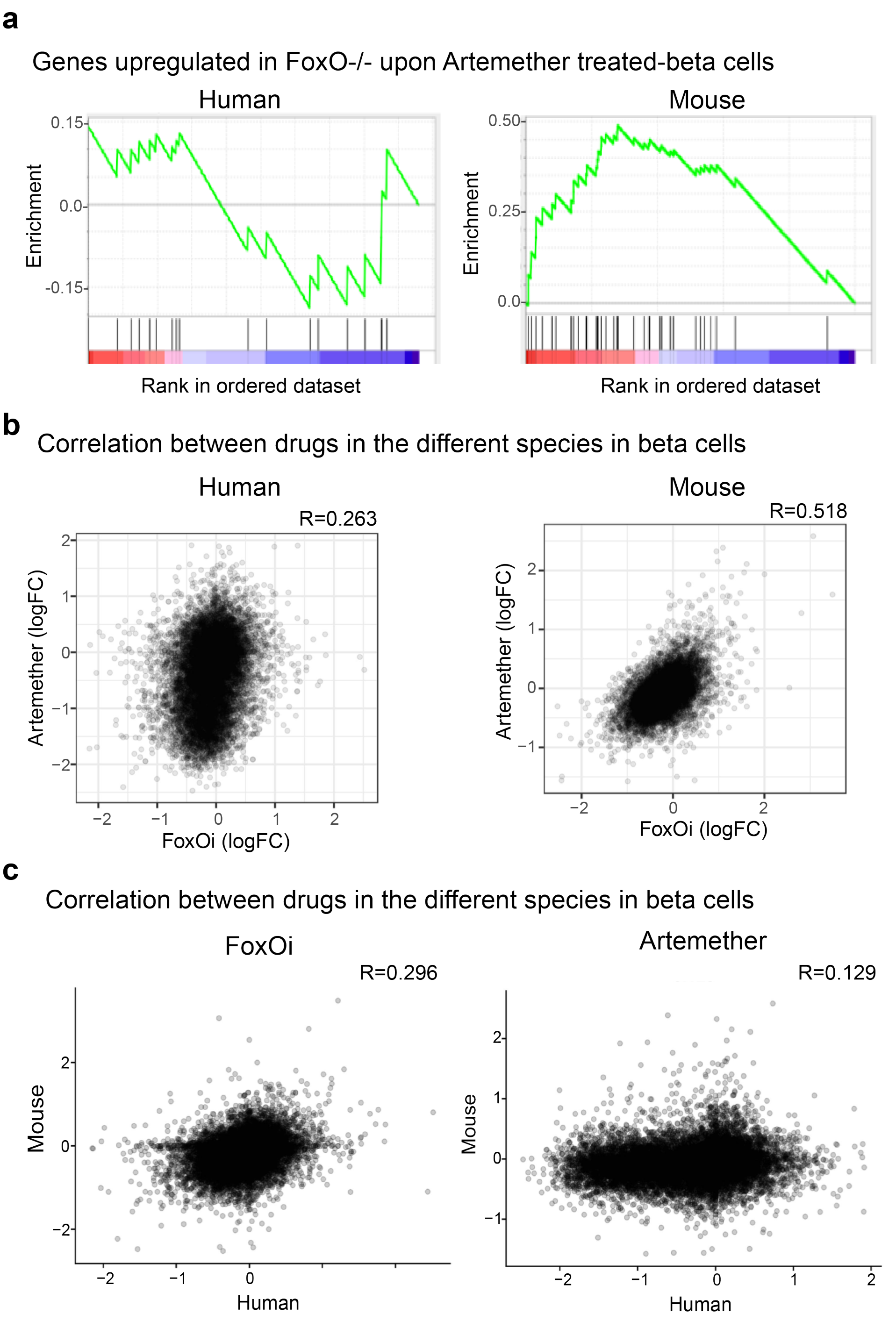

Figure S10: Artemether effects on beta cells are species dependent. (A) GSEA with the set of upregulated genes in FoxO$^{-/-}$ mice in beta cells from human and mouse islets treated with 10 μM artemether for 72 hr. **(B)** Correlation of gene expression changes induced by 10 μM FoxOi vs 10 μM artemether in human and mouse beta cells. (**C**) Gene expression changes induced by artemether treatment in beta cells have low correlation between mouse and human (R=0.129), changes induced by FoxOi have a higher correlation among species (R=0.296).

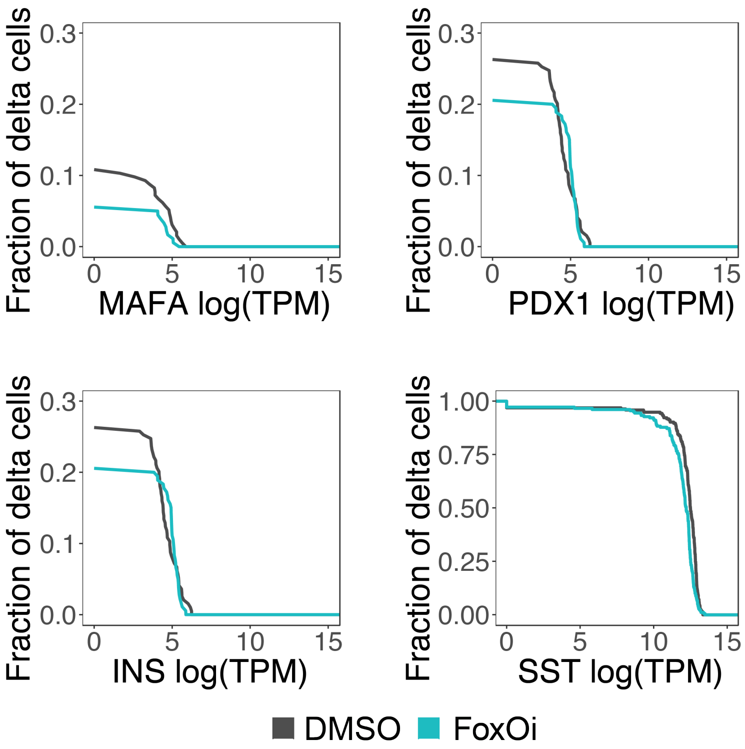

Figure S11: FoxOi effects in delta cells. Density plot of gene expression following DMSO and 1 μM FoxOi treatment for 72 hr for genes important in islet cell identity.
