# Supplementary material for: Single-cell RNA-seq with spike-in cells enables accurate quantification of cell-specific drug effects in pancreatic islets
Source: Genome Biol. 2020 May 6;21:106. doi: 10.1186/s13059-020-02006-2 (PMC7201533; doi:10.1186/s13059-020-02006-2)
Supplement: Supplementary file 10 — Additional file 10. Review history. [file 13059_2020_2006_MOESM10_ESM.docx]

Review History

**First round of review**

**Reviewer 1**

**Are you able to assess all statistics in the manuscript, including the appropriateness of statistical tests used?**

No, I do not feel adequately qualified to assess the statistics.

**Comments to author:**

This work addresses important questions. One is that single cell analysis of pancreatic islets has been confounded by the presence of extremely abundant transcripts (INS, GCG) in all cells, presumably due to the release of RNA from dead cells. The authors show that this is more pronounced in droplet based methods, but is also variably apparent in sorted cells. They have spiked in external mouse and human cell-lines before droplet formation, and used this to create models that facilitate in silico removal of contaminating RNAs. They find that corrected profiles do not accurately classify endocrine cell types, and develop a machine learning algorithm to overcome this problem. This component of the manuscript has the potential to provide a general interest method, but it has some shortcomings.

First, the Authors fail to discern endocrine cell types until they carry out the downstream machine learning-based analysis, whereas previous studies have not only identified endocrine cell types but also defined heterogeneity within cell types. If the authors feel that previous analyses were essentially flawed, and that the current framework provides a more accurate representation of endocrine cells, this should be demonstrated through direct comparisons. But it does not make much sense to present a new method which, at least at first glance, fails to achieve the resolution that is reported by existing methods.

Second, authors state that they "establish the use of standardized reference cells as spike-in controls for accurate and robust dissection of single-cell drug responses". This is potentially a novel contribution. However, most analyses presented in this manuscript does not compare the corrected data with the uncorrected data, and it is therefore impossible to evaluate the improvement. These type of comparisons are essential.

The other important component of this study concerns the use of FOXO inhibitors, which expectedly cause dedifferentiation, and artemether, which causes dedifferentiation of mouse but not human alpha cells, and induces insulin in both species. This is a controversial topic, because others failed to replicate the original description. It is therefore crucial that the authors provide more compelling evidence that alpha cells activate insulin in mouse and human alpha cells, and offer a more complete description of the transcriptional changes in both species. In the current form, the analysis does little to solve the controversy.

Specific comments

1. Summary quality control statistics before and after data correction (distribution of nUMI, nGene, %Mito gene reads, etc…) are necessary. How many, and what type of reads are removed? does this affect clustering? how does correction change the number of genes and UMIs detected per cell?

2. The lack of independent clustering of the endocrine subpopulation in the tSNE plots is unexpected, because endocrine cell types are typically resolved with uncorrected data. It would be informative to compare the results of clustering before and after data correction. Data analysis might benefit from using more recent versions of Seurat (v2.0.1 used, v3 available) and clustering visualization methods (e.g. UMAP).

A point of concern is that the correction method might entail removing low abundant expression transcripts that are informative for cell type classification and clustering. -Page 15 states that "To correct expression values, contamination signatures were multiplied by fc and subtracted from each cells expression values (TPM). Negative values were set to zero, and values were scaled up to reflect TPM." Can the authors demonstrate that by applying this correction method they are not loosing valuable gene expression information?

3. In Figure 3 authors present a machine learning based cell type assignation method to classify the non-assigned "endocrine" cell cluster. Authors should present what percentage of all cells does this "endocrine" cell cluster represent. Also, authors do not discuss hetero or homo doublets encapsulated in the same droplet. Have these cells been filtered out from the analysis? How could these doublets affect the cell type assignment?

4. Figure 4 shows that FOXOi induces dedifferentiation of alpha and beta cells, but this seems quite expected. Does this analysis make any specific contributions?

5. As far as I can see the analysis of FOXOi and artemether exposure data is not explicitly described in the methods section. Have the authors used their spike-in decontamination method and carried out downstream analyses? All of this should be laid out more clearly. It does not help that the legends in Figure 2B are wrong, but more evidence is needed to assert that INS+ alpha cells should be thought of as alpha cells, rather than any other endocrine cell type.

6. Authors should comment this spike-in method in the context of other computational approaches such as SoupX (https://www.biorxiv.org/content/10.1101/303727v1) and DecontX (https://www.biorxiv.org/content/10.1101/704015v1).

7. Page 9, line 21: "In mouse, the beta cell specific genes Ins1, Igf1r, Pdx1, Nkx6-1, Nkx2-2, Iapp, Foxo1, Abcc8 and Slc2a2 were all upregulated, corresponding to an increase in 'beta cell expression' and 'beta-cell development' specific gene sets." Where is this gene set enrichment analysis presented?

8. Page 11: "Artemether modulates GABA signalling, and expression changes by GABA and artemether are correlated (Fig. 6A)"

Authors do not present any transcriptional changes in GABA signaling pathway genes at the single cell level after GABA or Artemether treament, or do not offer any other transcriptional changes that could provide additional insights into the mechanism of actions of these agonists. This is in contrast with the transcriptional changes observed after Artemether treatment in their previous paper (Li et al. 2017: " Looking for changes in specific genes related to the compounds' mechanism, we found that GABRB3 and GABRG2 were strongly upregulated in artemether-treated α cells"). Do the authors detect these or other similar changes in their current scRNAseq datasets?

9. Misc. methodological issues:

-	Given the controversy concerning the reproducibility of agonist effects, the methods describing experimental treatment of islets should be much more detailed: provider, catalog number, lot number, form or preparation, storage and handling should be clearly stated for all used compounds (e.g. now missing for GABA and FOXOi).

-	More details on culture settings should be provided: antibiotics if any, provider, catalog number of the FBS used, etc.

-	Page 14 "Ambiguous cells were removed from the analysis" How many cells were removed? A workflow of all the filtering and quality controls illustrating how many cells have been removed from the analysis would be appreacited.

-	Page 15: "After decontamination, we noticed that one human sample (GABA in donor II) captured a large portion of total cells (21,978 of 60,054 human cells) but had very low quality (Fig. S3F) and higher contamination after correction (Fig. 2C). Since we only expect roughly 10,000 cells from each sample this case was deemed an outlier and excluded from further analysis, reducing the number of human cells to 38,076 (including 1,778 spike-ins), which together with the 35,333 mouse cells (including 1,420 spike-ins) resulted in a total number of 73,409 cells."

There is no figure S3F to illustrate the low quality of the sample. Authors do not present any quality control statistics or filtering steps. Additional clarification should be provided on why this sample has been removed from the analysis and why it presents higher contamination after correction.

-	Page 14: "In preparation of each single-cell RNA-seq run, 100 µl of the methanol fixed cells were supplemented with 900 µl ice-cold PBS slowly administered and" This particular way of rehydrating methanol fixed cells has been shown to degrade RNA (https://www.ncbi.nlm.nih.gov/pmc/articles/PMC6050658/). Can authors clarify if this could affect their current correction method.

Overall the text has several spelling mistakes and ambiguous sentences. Figures contain errors, captions are missing or legends point to non existing figures, such as:

Figure 3A and S4C: labels for gamma and delta cells seem to be mistaken

Figure S4G: clusters 7 and 10 with spike-ins cells: figure not described or captioned in the text. Related to this in Figure 3C Predicted cell type rows 7 and 11 are not explained in legend or main text.

No caption or explanation for figure S5A in main text.

Figure 5B: legend, both groups are labelled as INS+alpha cells

There is a caption for Figure 6C in page 11, but there is no figure

There is a legend for figure S9G, but there is no figure S9G.

**Reviewer 2**

**Are you able to assess all statistics in the manuscript, including the appropriateness of statistical tests used?**

No, I do not feel adequately qualified to assess the statistics.

**Comments to author:**

In this work, the authors demonstrate the usefulness of spike-in cells in removing background contamination from single-cell RNA-seq data generated using a droplet-based platform.

The authors then apply this model on rather impressive sc-RNA datasets of mouse and human pancreatic islet cells subjected to three different treatments (FoxOi, artemether, and GABA). The results illustrate how each of these treatments influence cell identity, and in the case of artemether reveal unexpected species-specific effects. The findings are validated using complementary bioinformatic approaches, but not by additional experiments.

The paper is well written and I enjoyed reading it. It addresses an important technical concern and advances current understanding of the biology of pancreatic islet cells. However, the optimal use of the spike in cell method involves sequencing the spike in cells in isolation as an additional library (as the signature of contamination was determined by comparing contaminated spike-ins to clean references). The costs of such additional sequencing run using the 10x genomics platform might currently be prohibitive for many smaller labs.

Major points:

1. The usefulness of the spike-in method is demonstrated on a single dataset, which arguably represents a potentially severe case of cross-contamination: the tissue is digested enzymatically,

and the cells express important transcripts of interest at very high levels. How much cross-contamination would be detected in more typical samples, such as the human-mouse datasets produced by 10x genomics?

https://support.10xgenomics.com/single-cell-gene-expression/datasets/3.0.0/hgmm_10k_v3

2. The authors find up to 20% of contamating reads, however, in many datasets from the 10x Genomics platform, the fraction of reads found in cells is much higher, even around 95%. Is there correlation between the degree of detected contamination (Figure 1E) and the fraction of reads outside cells, as reported in the Cellranger html summary files?

3. In essence, the spike-in method is based on quantifying the amount of cell-free ambient RNA in relation to cellular transcripts. However, I wonder if the use of spike-in cells is strictly necessary? Would it be possible to determine the contamination signature by just studying the data from the "empty" droplets that are normally discarded by the Cellranger pipeline?

4. Figure 2 C/D, Figure 4A (and 5A+D) - no levels of statistical significance are presented

Minor points:

1. Figure 1D colour key is missing

2. Figure 1G X/Y axis units are missing.

3. Figure 1H no color key is presented, although in the figure legend it is stated that the values shown are log TPM

4. In addition, I think the readers would benefit from a more detailed figure legend: at first glance, it is not obvious what labels "Human 1 and 2" and "Mouse 1 and 2" signify. How are the columns ordered?

5. Figure 1H: some ot the genes that are expressed relatively highly in isolated mouse reference cells, such as ARHGDIB and SNHG25 seem to be detected at much lower levels in those mouse spike ins that were mixed among human islet cells. What causes this? Is it due to the presence of contaminating counts from the islet cell transcriptome?

6. Figure S4 some text is too small

7. Page 15 line 18 : figure S3F is cited, but no such panel exists

8. It would be interesting to compare t-SNE and clustering (Figure 3A) with a similar analysis using uncorrected data

9. Some of the results and figures are actually introducted in the Discussion section (page 11 lines 56-57). I would recommend moving them to the Results section in the interest of clarity.

**Authors Response**

**Point-by-point responses to the reviewers’ comments:**

*We thank the reviewers for their constructive comments; in addressing them, we have significantly strengthened the manuscript in the revised version. A pdf version of this response including figures is attached as supplementary information.*

*Overall, in the course of the revision, we have made the following improvements to the manuscript:*

*- We now added a comparison of our spike-in based decontamination method to SoupX, a method assessing contaminating transcripts from otherwise empty droplets.*

*- We now added a computational approach for removing doublets based on deviations from cell-type-specific numbers of expressed genes. We show that the overall biological findings did not change with this removal of potential doublets.*

*- We added a third human donor after a time-course of artemether treatment of 36 and 72 hours. This further strengthened the evidence that 72 hours of artemether treatment increases the number of alpha cells that express insulin and does not decrease insulin expression in human beta cells.*

*- We now added supplementary tables containing overall gene expression changes, cell numbers from each sample, gene set enrichment analysis and statistical tests.*

*Please find our detailed point-by-point responses below.*

Reviewer reports:

Reviewer #1: This work addresses important questions. One is that single cell analysis of pancreatic islets has been confounded by the presence of extremely abundant transcripts (INS, GCG) in all cells, presumably due to the release of RNA from dead cells. The authors show that this is more pronounced in droplet based methods, but is also variably apparent in sorted cells. They have spiked in external mouse and human cell-lines before droplet formation, and used this to create models that facilitate in silico removal of contaminating RNAs. They find that corrected profiles do not accurately classify endocrine cell types, and develop a machine learning algorithm to overcome this problem. This component of the manuscript has the potential to provide a general interest method, but it has some shortcomings.

*We thank the referee for the general interest in our study and address the points described as shortcomings below.*

First, the Authors fail to discern endocrine cell types until they carry out the downstream machine learning-based analysis, whereas previous studies have not only identified endocrine cell types but also defined heterogeneity within cell types. If the authors feel that previous analyses were essentially flawed, and that the current framework provides a more accurate representation of endocrine cells, this should be demonstrated through direct comparisons. But it does not make much sense to present a new method which, at least at first glance, fails to achieve the resolution that is reported by existing methods.

*For most cells, we do discern endocrine cell types directly from sequencing data, and only for a small subset of cells initially labeled ‘endocrine’ we apply the machine learning approach to predict their identity. In our detailed answer below we describe how drug treatment, extended ex vivo culture, 10X technology and large cell numbers might all contribute to the observed t-SNE visualizations.*

Second, authors state that they "establish the use of standardized reference cells as spike-in controls for accurate and robust dissection of single-cell drug responses". This is potentially a novel contribution. However, most analyses presented in this manuscript does not compare the corrected data with the uncorrected data, and it is therefore impossible to evaluate the improvement. These type of comparisons are essential.

*Most of the contaminating reads correspond to the genes with highest expression in islet cells (Fig 1G), and most abundant contaminating genes are insulin and glucagon (Fig. 1H). Our data correction removes the insulin and glucagon contamination, resulting in better correlation of spike-in transcriptomes to external references (Fig. 2C) and in the presence of insulin-negative and glucagon-negative islet cells (Fig. 2E, S3).*

*Response Figure 1: Correlation of alpha and beta cell transcriptomes in raw data and after correction for decontamination for human islet samples.*

*The presence of contaminating transcripts further makes alpha and beta cells appear more similar to each other (i.e. the expression of contaminating glucagon in beta cells makes them more similar to alpha cells). Following decontamination, we observe lower correlation between alpha and beta cell transcriptomes (Response Fig. 1).*

*For all of these reasons, we have analyzed the decontaminated data set throughout the manuscript.*

The other important component of this study concerns the use of FOXO inhibitors, which expectedly cause dedifferentiation, and artemether, which causes dedifferentiation of mouse but not human alpha cells, and induces insulin in both species. This is a controversial topic, because others failed to replicate the original description. It is therefore crucial that the authors provide more compelling evidence that alpha cells activate insulin in mouse and human alpha cells, and offer a more complete description of the transcriptional changes in both species. In the current form, the analysis does little to solve the controversy.

*We do not claim to solve the controversy with this data set, but provide further evidence for transcriptional changes with the compounds in primary human and mouse islets in the absence of genetic lineage tracing reporters. To the best of our knowledge, dedifferentiation of alpha and beta cells treated with Foxo inhibitor has never been described before. The original publication (Nagashima et al. Mol. Pharmacol. 2010) described increased glucose tolerance in db/db mice treated with FoxOi due to suppression of hepatic gluconeogenesis. Later it was shown that the compound can improve reprogramming of delta cells to beta cells following near complete beta cell ablation (Chera et al. Nature 2014). No detailed transcriptional studies of the compound’s effect on islet cells have been published.*

*To provide a complete description of the cell-type specific transcription changes, we now include Supplementary table 1 reporting all differentially expressed genes with a q-value < 0.1.*

Specific comments

1. Summary quality control statistics before and after data correction (distribution of nUMI, nGene, %Mito gene reads, etc…) are necessary. How many, and what type of reads are removed? does this affect clustering? how does correction change the number of genes and UMIs detected per cell?

*Distributions of nUMI are shown in Fig. S4F/M, it is not possible to determine the nUMI after the correction because correction is performed on TPM-normalized data. The number of genes before and after correction is presented in the following panel, showing that the number of genes removed is minimal. Data correction improved the clustering of the different cell populations, as described below.*

Response Figure 2: Distributions of number of genes in raw (black) and corrected (red) data for each cell type.

2. The lack of independent clustering of the endocrine subpopulation in the tSNE plots is unexpected, because endocrine cell types are typically resolved with uncorrected data. It would be informative to compare the results of clustering before and after data correction. Data analysis might benefit from using more recent versions of Seurat (v2.0.1 used, v3 available) and clustering visualization methods (e.g. UMAP).

A point of concern is that the correction method might entail removing low abundant expression transcripts that are informative for cell type classification and clustering. -Page 15 states that "To correct expression values, contamination signatures were multiplied by fc and subtracted from each cells expression values (TPM). Negative values were set to zero, and values were scaled up to reflect TPM." Can the authors demonstrate that by applying this correction method they are not loosing valuable gene expression information?

*We performed cell population clustering using Seurat v2.0.1, the same version used in most published islet datasets. We believe that several factors contribute to the observed tSNE plot appearance:*

*1) In contrast to all published datasets, here we treated islets for 72 h with compounds. These compounds were selected based on altering identity of some cell populations, making some populations look more similar within each other.*

*2) The compound treatment required extended ex vivo culture, which may have an overall impact on islet transcriptomes.*

*3) Single-cell sequencing using 10X allows for sequencing of high number of cells however, at a lower depth than other methods, such as sorting followed by Smart-Seq2. Therefore, low cluster resolution might be inherent to the use of 10X. The one published islet 10X dataset (Xin et al. Diabetes, 2018; Dominguez Gutierrez et al. Endocrinology 2018) shows similar resolution of endocrine cell types as our dataset.*

*Response Figure 3: published islet single cell transcriptome dataset using 10X technology. (left panel from Xin et al. Diabetes, 2018; right panel from Dominguez Gutierrez et al. Endocrinology 2018)*

*4) Finally, the size of the dataset consisting of over 95,000 cells allows for detection of more cells that are “intermediate” between cell types than in the previously published datasets. In the tSNE plot (Fig. 3A) cells from both donors and all treatments are presented. Plotting smaller datasets, for each individual treatment and donor increases cluster resolution for endocrine cell types.*

*Response Figure 4: tSNE-plots of human endocrine cells, separated by donor and treatment*

3. In Figure 3 authors present a machine learning based cell type assignation method to classify the non-assigned "endocrine" cell cluster. Authors should present what percentage of all cells does this "endocrine" cell cluster represent. Also, authors do not discuss hetero or homo doublets encapsulated in the same droplet. Have these cells been filtered out from the analysis? How could these doublets affect the cell type assignment?

*Most cells are assigned to their cell type based on the expression of only one high expressing marker gene. The percentage of ’computationally predicted‘ cells is very low and significantly below 20% for all cell types (Fig. S5B/C). However, here we present a zoom-in into the quantification of the percentage of these cells among alpha and beta cells. Below we provide a ‘zoom in’ to quantify percentages of computationally predicted cell types.*

*Response Figure 5: Quantification of the percentages of cells originally labeled ‘endocrine’ that were assigned to specific cell types computationally.*

*We further show that the main findings on insulin induction by artemether hold true also when only considering cells assigned to endocrine cell types and not taking into account computationally predicted cell types (Fig. S7B).*

*Homo or hetero doublets are typically identified by having a higher gene number than single cells. However, in our dataset there is no clear cut-off to define doublets, rather a continuous distribution of number of genes vs number of UMIs. In order to study whether doublets could affect cell type assignment, we plotted the cell class probability vs. the number of genes. The cells with lower class probabilities actually have a lower number of genes, indicating that they are less likely to be doublets. Therefore, in the original submission we had not implemented any doublet filtering.*

*Response Figure 6: Doublet filtering. (A) Distribution of number of genes vs. number of UMIs. (B) Distribution of cell type probability vs. number of genes.*

*In the revised version, we have now included bioinformatic doublet removal after cell type assignment. We based this filter on the rationale that singlets should have a high class probability for only one class whereas doublets have high class probability for more than one class. This is in line with current tools for doublet filtering. We thus calculated the ratio between the highest and second highest class probability for each cell, and removed all cells with lower than 3-fold difference as doublets. In addition, we implemented a second filter for doublets based on the number of genes per cell (Ngenes). For each cell type and sample, we identified the expected Ngenes as the value of Ngenes with the maximal density. Next, we removed all cells with Ngenes greater than twice the expected Ngenes of the respective cell type and sample. This filtering is specific for each cell type and treatment and is based on gene number and class probability.*

*The overall biological findings did not change after double filtering.*

4. Figure 4 shows that FOXOi induces dedifferentiation of alpha and beta cells, but this seems quite expected. Does this analysis make any specific contributions?

*Beta cell dedifferentiation in mice has previously been described following genetic knock-out of the transcription factor FoxO1 (Talchai et al, 2012).*

*The selective FOXO1 inhibitor has been shown to improve fasting glycemia in diabetic db/db mice by reduced hepatic gluconeogenesis through the reduction of hepatic G6Pase and PEPCK (Nagashima et al, 2010). Furthermore, it was shown that the compound can improve reprogramming of delta cells to beta cells following near complete beta cell ablation (Chera et al. Nature 2014). No detailed studies of the compound’s effect on islet cells have been published.*

*In this manuscript, we hypothesize that the FoxO inhibitor can recapitulate dedifferentiation following genetic knock-out of FoxO transcription factors. Our observation that the FoxO inhibitor can pharmacologically model islet cell dedifferentiation has many important implications for islet biology:*

*- We are the first to show that pharmacological FoxO inhibition results in dedifferentiation of mature islet cells ex vivo.*

*- We show that the dedifferentiation process occurs with fast kinetics.*

*- To the best of our knowledge, effects of FoxO inhibition on alpha cell transcriptomes have not been characterized before, neither with inhibitors nor in genetic models.*

*- Our findings suggest the application of the FoxO inhibitor in screens for compounds and genetic factors that modulate islet cell dedifferentiation processes.*

5. As far as I can see the analysis of FOXOi and artemether exposure data is not explicitly described in the methods section. Have the authors used their spike-in decontamination method and carried out downstream analyses? All of this should be laid out more clearly. It does not help that the legends in Figure 2B are wrong, but more evidence is needed to assert that INS+ alpha cells should be thought of as alpha cells, rather than any other endocrine cell type.

*All data have been processed through a bioinformatics pipeline of decontamination, cell type assignment and doublet removal as described in the methods section.*

*We apologize for the labeling mistake in Fig. 5B, which has now been corrected.*

*Regarding the robustness of alpha cell assignment and prediction, we show that findings on increased insulin expression are also observed when only taking into account the alpha cells assigned solely based on high glucagon expression without considering computational cell type prediction (Fig. S7B). The increase in insulin positive alpha cells further holds up over all ranges of alpha cell probability from the computational predictor (S7C), and in islets from donors showing different insulin/glucagon and alpha/beta cell ratios (S7E).*

6. Authors should comment this spike-in method in the context of other computational approaches such as SoupX (https://www.biorxiv.org/content/10.1101/303727v1) and DecontX (https://www.biorxiv.org/content/10.1101/704015v1).

*We have added a comparison to SoupX for removing contaminating reads. In order to compare the performance of the two methods, we used the same method as presented in Figure 2C, using Pearson correlation of spike-in transcriptomes to externally reference transcriptomes from the same cells. Our spike-in based decontamination method resulted in higher correlation to the reference than the empty-droplet based SoupX method (Fig. S3B). This comparison is now included in Fig. S3B.*

7. Page 9, line 21: "In mouse, the beta cell specific genes Ins1, Igf1r, Pdx1, Nkx6-1, Nkx2-2, Iapp, Foxo1, Abcc8 and Slc2a2 were all upregulated, corresponding to an increase in 'beta cell expression' and 'beta-cell development' specific gene sets." Where is this gene set enrichment analysis presented?

*GSEA analysis for the INS+ alpha cells was performed by selecting the significantly upregulated genes in INS+ alpha cells vs. INS- alpha cells and using Enrichr to identify enriched pathways, from which regulation of gene expression and beta-cell development were identified in BioPlanet 2019 and Reactome 2016. We have now added a supplementary table including GSEA for alpha Ins+ cells, as well as for alpha and beta cells treated with Artemether and FoxOi.*

8. Page 11: "Artemether modulates GABA signalling, and expression changes by GABA and artemether are correlated (Fig. 6A)"

Authors do not present any transcriptional changes in GABA signaling pathway genes at the single cell level after GABA or Artemether treament, or do not offer any other transcriptional changes that could provide additional insights into the mechanism of actions of these agonists. This is in contrast with the transcriptional changes observed after Artemether treatment in their previous paper (Li et al. 2017: " Looking for changes in specific genes related to the compounds' mechanism, we found that GABRB3 and GABRG2 were strongly upregulated in artemether-treated α cells"). Do the authors detect these or other similar changes in their current scRNAseq datasets?

*We do not observe upregulation of GABA receptor subunits in the current dataset. Of all the genes observed previously to be changes with artemether treatment in alpha cells, only some of the genes follow the same trend as in the original publication. Different coverage and detection limits between the originally used Smart-Seq v2 method and the 10X technology used in the current study may limit the possibility of detecting lowly expressed genes, and we cannot rule out donor-to-donor differences.*

*Response Figure 7: Transcription changes in alpha cells treated with artemether for 72 h for genes previously described as affected by the compound.*

9. Misc. methodological issues:

- Given the controversy concerning the reproducibility of agonist effects, the methods describing experimental treatment of islets should be much more detailed: provider, catalog number, lot number, form or preparation, storage and handling should be clearly stated for all used compounds (e.g. now missing for GABA and FOXOi).

*We now added these details to the Methods sections.*

- More details on culture settings should be provided: antibiotics if any, provider, catalog number of the FBS used, etc.

*We now added these details to the Methods sections.*

- Page 14 "Ambiguous cells were removed from the analysis" How many cells were removed? A workflow of all the filtering and quality controls illustrating how many cells have been removed from the analysis would be appreacited

*Ambiguous cells refer to those which have a high number of reads for both human and mouse cells, indicating that they are doublets between islet samples and the mouse spike-ins. As can be seen in Fig 1D and S1B the number of cells identified as “ambiguous” is low. We have now rewritten the text to more clearly describe these droplets, and added a color code to Fig1D and S1B for better identification of the ambiguous cells.*

*Furthermore, we have included a supplementary table describing all cells in each sample, and included a figure describing the full data processing workflow (Fig. S6).*

- Page 15: "After decontamination, we noticed that one human sample (GABA in donor II) captured a large portion of total cells (21,978 of 60,054 human cells) but had very low quality (Fig. S3F) and higher contamination after correction (Fig. 2C). Since we only expect roughly 10,000 cells from each sample this case was deemed an outlier and excluded from further analysis, reducing the number of human cells to 38,076 (including 1,778 spike-ins), which together with the 35,333 mouse cells (including 1,420 spike-ins) resulted in a total number of 73,409 cells."

There is no figure S3F to illustrate the low quality of the sample. Authors do not present any quality control statistics or filtering steps. Additional clarification should be provided on why this sample has been removed from the analysis and why it presents higher contamination after correction.

*We apologize for the mistake in the text, which should have referred to Fig. S4F.*

*Several data points shown the low quality to the sample from GABA treated islets from human donor II:*

*- The sample has a low number of UMIs (Fig. 1F).*

*- The sample also has a low number of genes compared to other samples from the same islet batch (Response Fig. 2)*

*- It also has less reads in cells (~55%) compared to all the other samples, which typically have >85% of reads in cells.*

*- The sample has very poor cluster resolution (Response Fig. 4).*

*For all these reasons, we removed this sample from further analysis.*

- Page 14: "In preparation of each single-cell RNA-seq run, 100 µl of the methanol fixed cells were supplemented with 900 µl ice-cold PBS slowly administered and" This particular way of rehydrating methanol fixed cells has been shown to degrade RNA (https://www.ncbi.nlm.nih.gov/pmc/articles/PMC6050658/). Can authors clarify if this could affect their current correction method.

*The methanol fixed cells that we used as spike-ins for the islet runs were treated the same way as the spike-in cells that were used as “reference” spike-in cells that were sequenced separately. Having the same method for both the reference cells and the ones used in the islet sequencing, we can ensure that the methanol fixation protocol did not affect our correction method.*

Overall the text has several spelling mistakes and ambiguous sentences. Figures contain errors, captions are missing or legends point to non existing figures, such as:

Figure 3A and S4C: labels for gamma and delta cells seem to be mistaken

*Thank you for pointing out this mistake, which we have now corrected.*

Figure S4G: clusters 7 and 10 with spike-ins cells: figure not described or captioned in the text. Related to this in Figure 3C Predicted cell type rows 7 and 11 are not explained in legend or main text.

*We have now added a clarification in the figure legend and methods, describing that we have not assigned specific cell types to cells found in the clusters labeled with numbers.*

No caption or explanation for figure S5A in main text.

*We have now added a reference to figure S5A in the main text.*

Figure 5B: legend, both groups are labelled as INS+alpha cells

*We have now corrected the labeling.*

There is a caption for Figure 6C in page 11, but there is no figure

*We have now corrected to refer to Fig. 6B.*

There is a legend for figure S9G, but there is no figure S9G.

*We have now added a Fig. S10 describing effects of the FoxO inhibitor on delta cells.*

Reviewer #2: In this work, the authors demonstrate the usefulness of spike-in cells in removing background contamination from single-cell RNA-seq data generated using a droplet-based platform.

The authors then apply this model on rather impressive sc-RNA datasets of mouse and human pancreatic islet cells subjected to three different treatments (FoxOi, artemether, and GABA). The results illustrate how each of these treatments influence cell identity, and in the case of artemether reveal unexpected species-specific effects. The findings are validated using complementary bioinformatic approaches, but not by additional experiments.

The paper is well written and I enjoyed reading it. It addresses an important technical concern and advances current understanding of the biology of pancreatic islet cells. However, the optimal use of the spike in cell method involves sequencing the spike in cells in isolation as an additional library (as the signature of contamination was determined by comparing contaminated spike-ins to clean references). The costs of such additional sequencing run using the 10x genomics platform might currently be prohibitive for many smaller labs.

*We thank the referee for the positive comments on our manuscript.*

It is true that in order to optimally make use of our method, the spike-in cells must be sequenced individually. However, a single validated batch of spike-in reference cells can be used for numerous single cell sequencing experiments, and the number of added spike-in cells is flexible (5% in our case, but this could be scaled down further). Coordination between sequencing facilities to use generally accepted and validated reference cell batches, or inclusion of reference cells into single cell sequencing consumable kits would result only in a minimal cost increase for each individual single cell transcriptomics experiment.

Major points:

1. The usefulness of the spike-in method is demonstrated on a single dataset, which arguably represents a potentially severe case of cross-contamination: the tissue is digested enzymatically, and the cells express important transcripts of interest at very high levels. How much cross-contamination would be detected in more typical samples, such as the human-mouse datasets produced by 10x genomics?

https://support.10xgenomics.com/single-cell-gene-expression/datasets/3.0.0/hgmm_10k_v3

*We used the dataset of the classical experiment of mixing human and mouse cells to study single cells and discriminate doublets, in this case a 1:1 mixture of fresh frozen human HEK293T cells and mouse NIH3T3 cells (https://support.10xgenomics.com/single-cell-gene-expression/datasets/3.0.0/hgmm_10k_v3).*

*We estimated the contamination in this example dataset and in our cells and obtained comparable levels of contamination.*

*Response Figure 8:Percentage of contaminating reads in standard 10X Genomics HET293T/NIH3T3 mixing experiments (left panels) and in our dataset (right panel).*

2. The authors find up to 20% of contamating reads, however, in many datasets from the 10x Genomics platform, the fraction of reads found in cells is much higher, even around 95%. Is there correlation between the degree of detected contamination (Figure 1E) and the fraction of reads outside cells, as reported in the Cellranger html summary files?

*There seems to be a trend towards a higher percentage of reads in cells (=lower reads outside the cells) and higher contamination of the sample.*

*Response Figure 9: Scatter plot for reads in cells vs. percent contamination. The point marked in the red square corresponds to the GABA sample from donor II.*

3. In essence, the spike-in method is based on quantifying the amount of cell-free ambient RNA in relation to cellular transcripts. However, I wonder if the use of spike-in cells is strictly necessary? Would it be possible to determine the contamination signature by just studying the data from the "empty" droplets that are normally discarded by the Cellranger pipeline?

*We now included a comparison of our method to remove contaminating reads to SoupX (https://www.biorxiv.org/content/10.1101/303727v1), a method based on estimating contamination from otherwise empty droplets. In order to compare the performance of the two methods, we used the same approach as presented in Figure 2C. In short, we calculated the correlation of the human and mouse spike-ins in the human samples to their corresponding reference transcriptome and compared both methods. We observe higher correlation to the reference with the spike-in based method compared to SoupX (Fig. S3B).*

4. Figure 2 C/D, Figure 4A (and 5A+D) - no levels of statistical significance are presented

*We now added the corresponding statistics to the figures and also include a supplementary table with the p-values for all comparisons.*

Minor points:

1. Figure 1D colour key is missing

*We now added the color key to Fig. 1D and also Fig. S1.*

2. Figure 1G X/Y axis units are missing.

*We now added the axis units to Fig. 1G and S1.*

3. Figure 1H no color key is presented, although in the figure legend it is stated that the values shown are log TPM

*We now added the color key to Fig. 1H.*

4. In addition, I think the readers would benefit from a more detailed figure legend: at first glance, it is not obvious what labels "Human 1 and 2" and "Mouse 1 and 2" signify. How are the columns ordered?

*Fig. 1H shows the top and bottom 20 genes when comparing sample spike-ins to clean references. The columns are sorted by this difference. We have now edited the figure for clarity.*

5. Figure 1H: some ot the genes that are expressed relatively highly in isolated mouse reference cells, such as ARHGDIB and SNHG25 seem to be detected at much lower levels in those mouse spike ins that were mixed among human islet cells. What causes this? Is it due to the presence of contaminating counts from the islet cell transcriptome?

*Yes, when we compare the spike-ins in the islet sample vs the reference the spike-ins, some of the very highly expressed genes have a reduction in the expression. We believe that this is caused by the contamination coming from the islet cells taking up some reads in the spike-in cells, therefore making it seem that the expression for some genes appear lowered in the spike-ins.*

6. Figure S4 some text is too small

*We have now increased the font size for some of the text in Fig. S4*

7. Page 15 line 18 : figure S3F is cited, but no such panel exists

*We have now corrected this to correctly point to Fig. S4F.*

8 8. It would be interesting to compare t-SNE and clustering (Figure 3A) with a similar analysis using uncorrected data

*Below we present a tSNE plot with the raw and corrected data for both human donors for each treatment. In the tSNE representation we observed no evident improvement of clustering with the data correction.*

*We therefore assessed the improvement of cell clustering after decontamination with other tests. The presence of contaminating transcripts in the cells makes the alpha and beta cells appear more similar to each other (i.e. the expression of contaminating glucagon in beta cells makes them more similar to alpha cells), therefore its removal should make them look less similar. Comparing raw and corrected data of alpha and beta cell transcriptomes, we observe lower correlation of the different cell types after decontamination (Response Figure 1). These finding confirm the relevance of the computational workflow we developed.*

*Response Figure 10: t-SNE plots of raw and corrected data.*

9. Some of the results and figures are actually introducted in the Discussion section (page 11 lines 56-57). I would recommend moving them to the Results section in the interest of clarity.

*We now moved the introduction of the Drop-seq data from another donor to the results section, where we also now included 10X data from an additional donor.*

**Second round of review**

**Reviewer 1**

The authors have provided an improved version of this manuscript, although they have not directly addressed major points raised in the previous review. They have not properly benchmarked their method against an alternative state of the art option based on no spike-ins. Despite that cross-contamination is a major issue in most if not all scRNA-seq experiments, 10X chromium data can offer reasonable discrimination of major cell types in complex tissues such as pancreatic islets. Machine learning classifiers that ignore the simple presence/absence of most abundant genes could also be applied to 10X data. Furthermore, in this manuscript the machine learning approach was trained with 5-fold validation, but authors do not assess report model accuracy and just provide mean probabilities of the cells that are assigned to each cell type. It is therefore unclear if the spike-in/machine learning method represents an improvement. Clearly external spike-ins make sense in some settings, but does this manuscript meet standards to convince others to implement this particular method?

The authors also claim that artemether (and to some extent GABA, p. 23 and end of discussion) leads to an increase in the fraction of alpha cells that express insulin. Two papers in Cell from the authors claimed that artemether and GABA signaling modulators could be used for beta cell regeneration, but this was challenged by subsequent publications (e.g. Huising and Kaestner labs) who failed to replicate the original studies. The data from the current study is not very convincing. For example, data from a new sample in fig s8 shows that artemether increases insulin in alpha cells in 2 replicates and reduces insulin in 1 replicate at 72 hours, and increases insulin in alpha cells in 1 replicate and markedly reduces in 1 replicate at 36 hours. It is impossible to conclude from this that there is a stimulatory effect. One could instead conclude that the percentage of insulin expressing alpha cells is very variable. The dropSeq analysis in figure s8 is analyzed differently, but again the data that is shown is not very conclusive. The authors conclude from their results that the effects they observed explain the effects they had previously reported on glycemia: “Based on our and others’ studies it is clear that artemisinin and GABA, and FoxOi impact gene expression and cell identity in isolated pancreatic islets from mouse and human, and that they alter glucose tolerance in mice in vivo”. This is misleading, given that they have shown a small increase in the fraction of cells expressing small amounts of insulin with artemisin, and this has not been very reproducible (as mentioned previously). Furthermore there is a lack of any real data for GABA. Are these minor changes in transcriptional levels in a few cells? If so, can this affect glycemia? The authors, for example, have not documented more glucagon+/insulin+ cells after treatment.

**Reviewer 2**

The manuscript by Marquina Sanchez et al. describes a single-cell study of drug responses in pancreatic islet cells. The authors introduce a method for using spiked in cells to remove background contamination that is pervasive in droplet-based scRNA-seq datasets, and can be particularly problematic with cells that express high levels of cell-type specific transcript. Pancreatic cells represent one example, other relevant cases would probably include e.g. antibody-secreting B cells.

Upon the initial submission I raised some concerns that were mostly technical in nature. In the revised paper these concerns have been addressed satisfactorily. I still feel that the adoption of the spike-in method among researchers might ultimately be limited by practical concerns and the introduction of computational methods not requiring the use of additional reagents.

However, I do think that the method itself is sound and the authors have demonstrated its usefulness - and in the revised manuscript included all the necessary controls. Therefore, I would recommend considering this revised manuscript for publication.

**Authors Response**

**Point-by-point responses to the reviewers’ comments:**

Reviewer #1:

The authors have not addressed the major questions I had posed, and after reading the manuscript again, I am concerned that they are not being straightforward about the artemether and GABA data.

IN summary, this is a methods paper that addresses an important problem but does not provide proper benchmarking to convincingly show it represents an advance over the state of the art. The results with exposure to chemicals are not particularly novel or straightforward, and they do not settle a controversy either. They are insufficient to justify publication beyond the method.

It is worth remembering that two papers from Huising and Kaestner labs in Cell Metab have refuted two back to back papers from the current authors in Cell regarding artemisin and GABA

. The authors don't clearly acknowledge this controversy, even in the response to my comments in the review (they talked about FOXO inhibitors, which actually are not controversial) instead of artemether.

In short, I think this idea has value but I have some doubts about whether it will spark sufficient interest for Genome Biology and there are very weak points that will not go unnoticed in the pancreatic beta cell community. I realize that this uncomfortable because the other reviewer seemed more positive.

*We feel that reviewer 1’s criticism is not justified as we did address all points of the first round of submission (which were more specific); however, the revisions were not considered by the reviewer in the second round.*

*The reviewer claims that we have not done proper benchmarking to show that our method represents an advance over the state of the art. However, we benchmarked our method along multiple lines: First, we clearly show that in currently published single cell islet transcriptomes from different groups all cell types are described to express insulin and glucagon. We assume that the referee agrees with us that such pan-cell type expression of hormone genes does not reflect the underlying biology and thus must be caused by contamination. Second, we clearly show that the method we developed is capable of removing this contamination. Third, we benchmark the method extensively using the known transcriptomes of spike-in cells. Fourth, we show that computational decontamination using standard approaches makes alpha and beta cell transcriptomes less similar to each other, as is expected. Fifth, we benchmark our method against state-of-the-art purely computational methods (SoupX) that do not rely on spike-in cells, and find improved performance. We further identify that the estimation of contamination fraction (which is difficult to do without spike-in cells) is key to this improved performance. In summary, we believe that we thoroughly benchmarked our method along multiple lines, and thereby comprehensively addressed all questions by this referee. Given that the reviewer neither criticizes our experiments nor proposes alternative benchmarking experiments, we fail to follow the reviewer’s claim that our manuscript does “not provide proper benchmarking”.*

*The second point raised by reviewer 1 concerns the controversy on the effects of artemether and GABA. He/she states that we do not clearly acknowledge this controversy, which is not true as we immediately introduce it after the first mention of these compounds in the introduction (“However, the mechanisms of artemether and GABA have been controversial, as two recent studies have not detected an increase in alpha-cell derived beta cells using lineage tracing models [8, 40]”). Also in the response letter, we refer to the controversy (“We do not claim to solve the controversy with this data set, but provide further evidence for transcriptional changes with the compounds in primary human and mouse islets in the absence of genetic lineage tracing reporters.”) and only later answer to the referee’s in our view unjustified point on the apparent obviousness of the Foxo inhibitor effects. We thus do extensively acknowledge the controversy, and in this second revision have rewritten the discussion to further reflect this point. Our data on treatment of human and mouse pancreatic islets are novel (a similar dataset has not been published in the meantime). We do agree that our results do not settle the controversy in full, but nevertheless they provide important additional information. In particular, the observed species differences of compound effects in human and mouse beta cells and different detection methods may indeed contribute to explaining some of the earlier results. Again, we fail to understand (from the minimal reviewer comments) how, given that the reviewer seems to appreciate the significance of the controversy, our results are deemed to be not novel or relevant.*

Reviewer #2: The manuscript by Marquina Sanchez et al. describes a single-cell study of drug responses in pancreatic islet cells. The authors introduce a method for using spiked in cells to remove background contamination that is pervasive in droplet-based scRNA-seq datasets, and can be particularly problematic with cells that express high levels of cell-type specific transcript. Pancreatic cells represent one example, other relevant cases would probably include e.g. antibody-secreting B cells.

Upon the initial submission I raised some concerns that were mostly technical in nature. In the revised paper these concerns have been addressed satisfactorily. I still feel that the adoption of the spike-in method among researchers might ultimately be limited by practical concerns and the introduction of computational methods not requiring the use of additional reagents.

However, I do think that the method itself is sound and the authors have demonstrated its usefulness - and in the revised manuscript included all the necessary controls. Therefore, I would recommend considering this revised manuscript for publication.

*We thank the referee for recommending our manuscript for publication. Regarding the raised concerns for practical considerations, we would like to point out that in other genomics methods like ChIP-seq, different species spike ins have become routine, commercially available and fundamental to normalize the data (Bonhoure et al. Genome Res. 2014, Orlando et al. Cell Rep., 2014, Chen et al. Mol. Cell. Biol. 2015, Gryder et al. Nature Protocols 2018, Blackledge et al. Mol. Cell 2020). Also, molecular spike-ins (ERCC) have been widely applied in bulk RNA-seq, to normalize data processing. Injection of fixed spike-in cells into a single- cell suspension is a simple procedure. While we agree that it complicates things over a purely computational correction method, we believe and actually show in the manuscript that the gain in accuracy is worth the extra effort.*
